# Unraveling axonal mechanisms of traumatic brain injury

**DOI:** 10.1186/s40478-022-01414-8

**Published:** 2022-09-21

**Authors:** Victorio M. Pozo Devoto, Valentina Lacovich, Monica Feole, Pratiksha Bhat, Jaroslav Chovan, Maria Čarna, Isaac G. Onyango, Neda Dragišić, Martina Sűsserová, Martin E. Barrios-Llerena, Gorazd B. Stokin

**Affiliations:** 1 Translational Neuroscience and Ageing Program, Centre for Translational Medicine, International Clinical Research Centre, St. Anne’s University Hospital, Brno, Czech Republic; 2 Biostatistics Department, International Clinical Research Centre, St. Anne’s University Hospital, Brno, Czech Republic; 3 Proteomics and Mass Spectrometry Core Lab, International Clinical Research Centre, St. Anne’s University Hospital, Brno, Czech Republic; 4 Division of Neurology, University Medical Centre, Ljubljana, Slovenia; 5 Department of Neurosciences, Mayo Clinic, Rochester, MN USA

**Keywords:** Axonal swellings, Traumatic brain injury, Calcium, Microtubules, Subcortical periodic cytoskeleton, Phosphoproteomics, Axonal transport

## Abstract

Axonal swellings (AS) are one of the neuropathological hallmark of axonal injury in several disorders from trauma to neurodegeneration. Current evidence proposes a role of perturbed Ca^2+^ homeostasis in AS formation, involving impaired axonal transport and focal distension of the axons. Mechanisms of AS formation, in particular moments following injury, however, remain unknown. Here we show that AS form independently from intra-axonal Ca^2+^ changes, which are required primarily for the persistence of AS in time. We further show that the majority of axonal proteins undergoing de/phosphorylation immediately following injury belong to the cytoskeleton. This correlates with an increase in the distance of the actin/spectrin periodic rings and with microtubule tracks remodeling within AS. Observed cytoskeletal rearrangements support axonal transport without major interruptions. Our results demonstrate that the earliest axonal response to injury consists in physiological adaptations of axonal structure to preserve function rather than in immediate pathological events signaling axonal destruction.

**Supplementary Information:**

The online version contains supplementary material available at 10.1186/s40478-022-01414-8.

## Introduction

Traumatic brain injury (TBI) is a major cause of disability and death [1]. As a consequence, the brain develops diffuse axonal injury (DAI) and focal enlargements within the axons known as axonal swellings (AS) [2, 3]. AS are more common in unmyelinated axons [4] and besides TBI, occur physiologically during aging [5], and in a range of pathological conditions from developmental disorders [6] to neurodegeneration [7]. Several studies have shown that pathology of the DAI involves several processes which may or may not be related to AS, such as Wallerian degeneration [8], mitochondrial dysfunction [9], aberrant protein accumulations [10] and proteases activation [11]. In addition, processes external to the neurons can also function as triggers of axonal collapse, including immune responses [12], changes in myelination [13] and energy deficiency [14].

Studies of AS report disruption [15], breakage or loss of microtubules [16] and increased neurofilament immunoreactivity [17]. Described cytoskeletal changes, coupled to observed aberrant accumulation of cargos, suggest that AS are the aftermath of impaired axonal transport [18, 19]. This hypothesis, however, is challenged by studies showing accumulation of cargos without cytoskeletal disruption [20] and AS lacking significant accumulation of cargos [21].

Since early descriptions of increased axonal Ca^2+^ levels following injury [22], the role of Ca^2+^ in axonal injury has been the subject of intense research. Ca^2+^ surge has been observed either within AS [23] or across the entire axon [24, 25]. Different sources have been proposed to underlie the observed Ca^2+^ increase: the extracellular milieu through mechanosensitive channels [26], voltage-gated Ca^2+^ channels [27] and the Na^+^-Ca^2+^ exchanger [28], and intracellularly through the ER Ca^2+^ stores [24]. Studies have also shown that attenuating Ca^2+^ increase after injury reduces axonal degeneration [25, 29]. Increased axonal Ca^2+^ levels have in fact been shown to affect cytoskeletal organization through several mechanisms involving microtubules stability [26], calpain activation [29] or calcineurin [24]. Apart from Ca^2+^, inhibition of ROCK [30] reduced swelling formation in response to axonal injury. This finding, together with observed RhoA activation following TBI [31], suggests roles of the RhoA/ROCK signaling in the AS formation.

To date, several experimental settings have been developed to study mechanisms of AS formation. Most of our current knowledge comes from in vitro models where neurites are stretched using dynamic substrate [28] or bent using pressurized air [32], magnetic tweezers [30] or media puffing [26]. However, some characteristics such as lack of discrimination between axonal or dendritic projections, or the use of neurons which are not fully mature, can be a hindrance to reproduce and elucidate the pathophysiology of TBI. Furthermore, omic approaches, apart from few exceptions [33, 34], have not been fully exploited to study axons under normal or pathological conditions. This can be attributed mainly to the difficulty in both the isolation of an exclusive axonal fraction and the collection of sufficient material to perform a reproducible analysis. The advancement of new approaches is fundamental to understand molecular mechanisms of the very first moments of the axonal response to injury and thus to shed light to the pathology of TBI.

We here developed a human mature neuron-based system to study the immediate effects of traumatic injury on the axonal structure and function. We also generated an algorithm which allowed us to assess the real-time dynamics of the AS formation and examine the role of Ca^2+^. The system offered the unique opportunity to describe the axonal proteome, identify its phosphorylation changes and predict the kinases that are activated and deactivated in the immediate axonal response to injury. Driven by these results, we used superresolution microscopy to characterize the earliest cytoskeletal changes taking place in the axon. Finally, we assessed transport dynamics to understand the functional consequences of the traumatic injury.

## Results

### Set-up of a novel axonal injury system

**Fig. 1:**
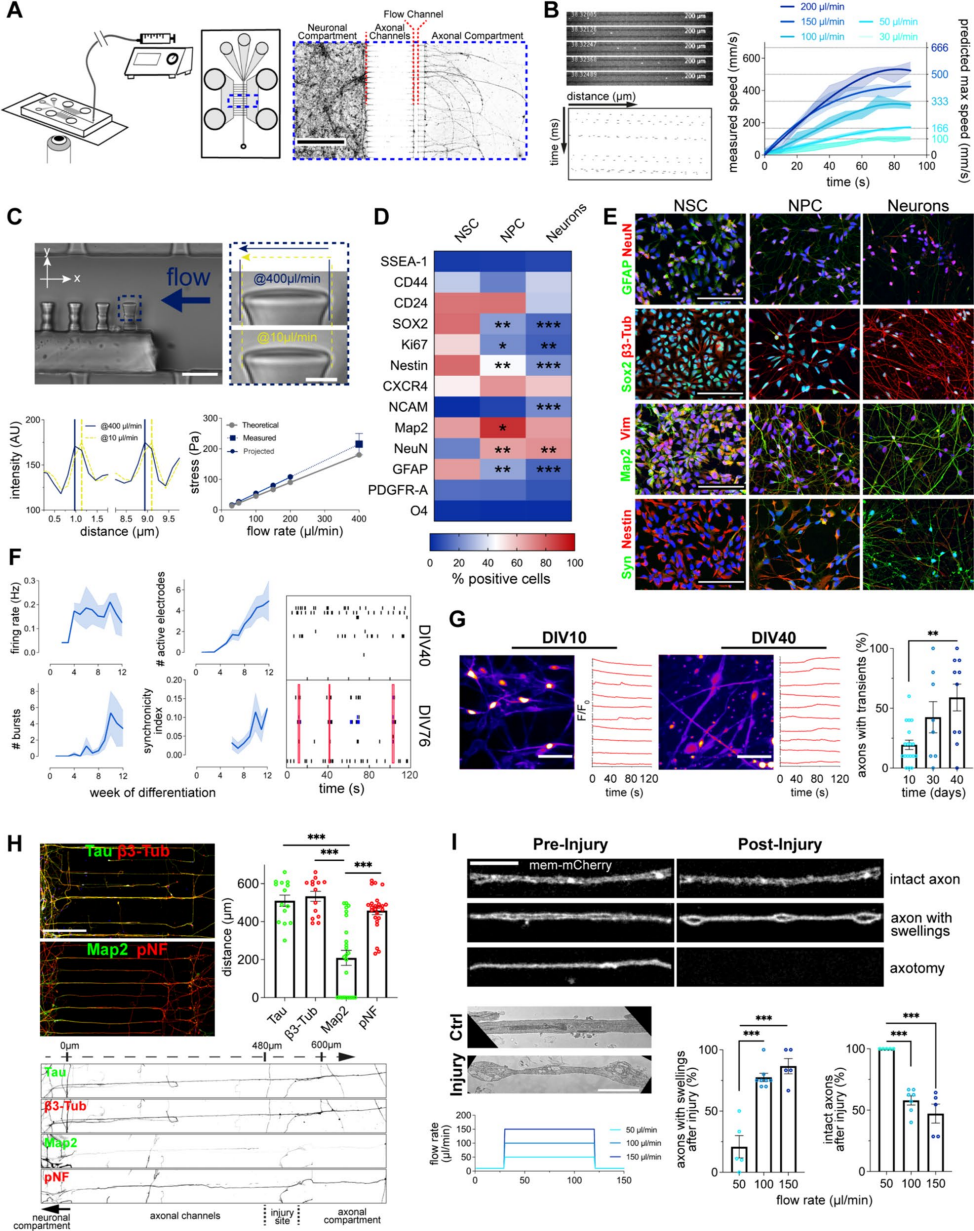
Set-up of a novel axonal injury system. **A** Schematic representation of the injury system. Image of microfluidic chamber compartments with WGA-stained neurons (scale bar: 500 µm). **B** Quantification of the speed of the media at different pump flow rates by tracking fluorescent beads in the flow channel (n = 3 independent chambers). **C** Calculation of the maximum stress applied in the flow channel by quantification of the urethane pillar bending (n = 3 independent chambers, scale bar: 20 µm, close-up: 3 µm). **D** and **E** Flow cytometry quantification (**D**) and immunofluorescence images (**E**) of the expression and localization of neuronal lineage markers through the differentiation stages from NSCs to mature neurons (FACS: n = 3, 250.000 cells/n; IF: n = 3, scale bar: 100 µm). **F** Electrophysiological activity during neuronal terminal differentiation (from DIV0 to DIV90, expressed in weeks) recorded on MEA plates. Right plots: representative raster plots of DIV40 and DIV76 recordings (n = 3, 4 wells/n). **G** Quantification of Ca^2+^ transients in cultures incubated with Fluo-4 AM through neuronal terminal differentiation (from DIV0 to DIV40) (n = 2, 5 recordings/n, scale bar: 50 µm). **H** Immunostaining against Tau, β3-Tub, Map2 and pNF (SMI31) of DIV40 neurons cultured in microfluidic chambers. Axonal and dendritic length measurements from the neuronal compartment to the axonal compartment (n = 3, 5–10 projections/n, scale bar: 200 µm). **I** Effects of different pump flow rates on AS generation and axotomy. Axons of mem-mCherry transduced neurons (DIV40) imaged before and after different pump flow rates for 90 s (n = 5, scale bar: 10 µm). TEM of axons in the flow channel (scale bar: 2 µm). Data are mean ± SEM (*p < 0.05, **p < 0.01, ***p < 0.001). Statistical comparisons were performed using one-way ANOVA followed by Dunnett’s multiple comparison test against NSCs group (**D**) or DIV10 group (**G**), or the Tukey’s multiple comparison test (**H** and** I**). See also Additional file 1: Fig. S1

To investigate the immediate axonal response to injury in real-time, we coupled a microfluidic chamber [35] harboring an exclusively axonal compartment to a syringe pump (Fig. 1A). In this system, the negative pressure applied by the pump produces hydrodynamic force in the flow channel, which intersects the axons perpendicularly. To validate the system, we used fluorescent beads to measure the speed of the media in the flow channel (Fig. 1B, Additional file 1: S1A, Additional file 2: Video S1). Pump flows between 30 and 200 µL/min produced maximum speeds similar to the ones predicted by the continuity equation and the finite element analysis. To estimate the stress to which axons are subjected, we placed urethane pillars into the flow channel and measured their displacement under the maximum pump flow of 400 µl/min (Fig. 1C, Additional file 1: Fig. S1A, Additional file 3: Video S2). The stress reached 200 Pa, which was comparable to the values obtained theoretically using finite element analysis.

We next examined differentiation, enrichment and maturity of the human neurons used in our system. At the mRNA level, early stage markers of differentiation showed a gradual decrease, while markers of neuronal maturity showed increased expression across the differentiation stages (Additional file 1: Fig. S1B). At the protein level, assessment of pluripotency, mesodermal, neuroblast, neural lineage, glial and neuronal markers by FACS showed neuronal enrichment and maturity of terminally differentiated neurons further supported by the immunostaining (Fig. 1D, E and Additional file 1: S1C, S1D). Electrical activity was first registered during the 3^rd^ week of maturation and continued increasing throughout the 12 weeks in culture (Fig. 1F), while the bursts first appeared during the 5th week followed by development of interneuronal connectivity. These neurophysiological parameters were accompanied by an increase in the prevalence and intensity of the Ca^2+^ transients throughout the 40 days of neuronal differentiation (Fig. 1G, Additional file 1: S1E, Additional file 4: Video S3).

We last tested whether projections at the site of injury correspond exclusively to the axons (Fig. 1H). Immunofluorescence showed β3-tubulin, tau and phosphorylated neurofilament heavy chain, but not MAP2 immunoreactivity at the intersection and beyond the flow channel. We finally labelled cellular membrane using palmitoylated mCherry (mem-mCherry) and quantified AS and axotomy at different flow rates (Fig. 1I). We identified 100 µL/min as the optimal flow rate to generate AS without significant axotomy. To confirm that mem-mCherry labelled AS indeed correspond to the actual AS, we performed TEM and observed focal enlargements of the axonal shafts as previously described (Fig. 1I).

### Waxing and waning behavior of the axonal swellings

**Fig. 2:**
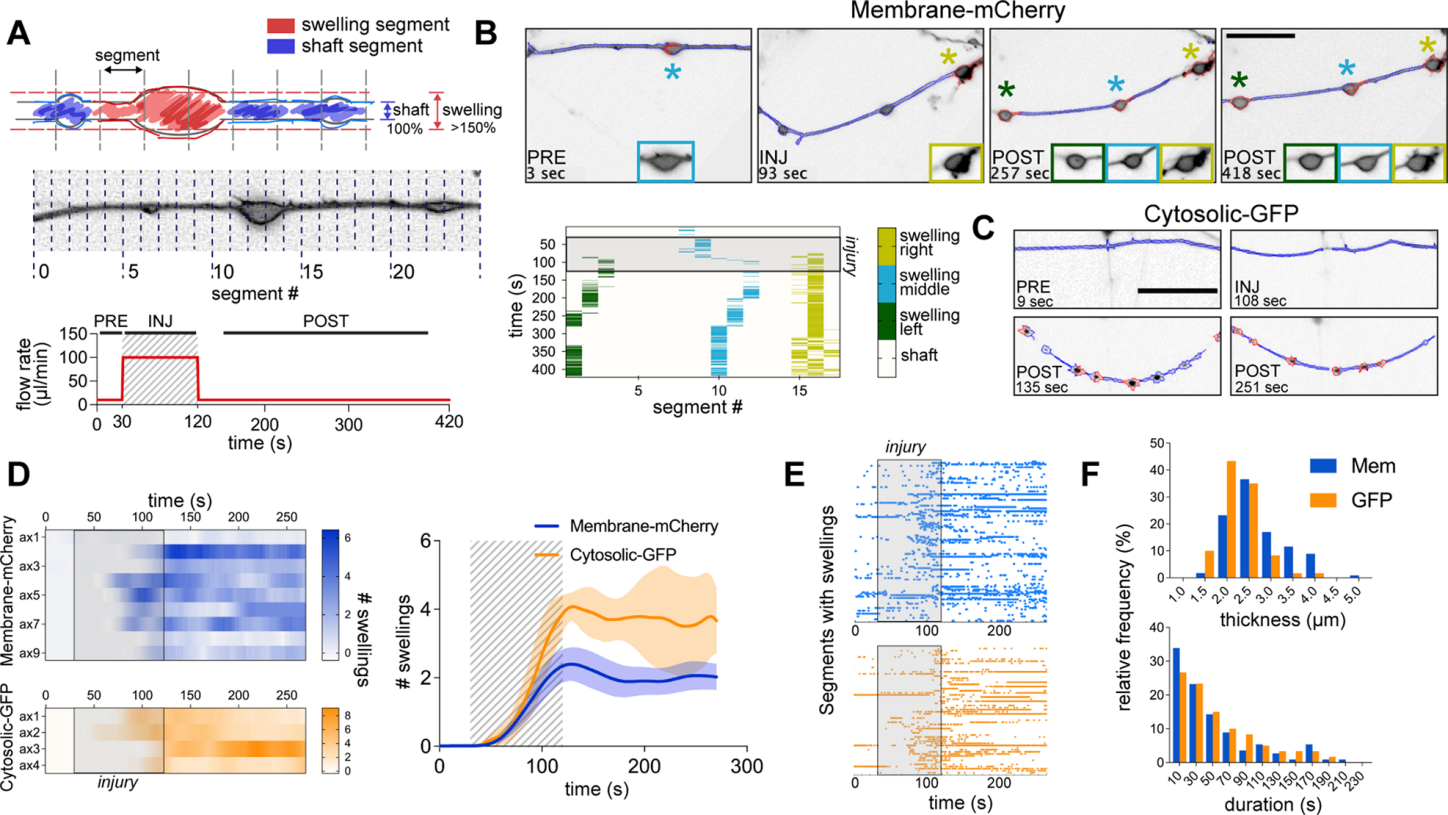
Wax and wane behavior of the axonal swellings. **A** Schematic representation of the analytical method and the injury protocol for the AS detection. The axon is divided into segments of equal sizes. For a swelling to be detected, the width of the axon in that segment has to be greater than 1.5 × of the median axonal shaft width in that frame. **B** AS tracking method of axons subjected to injury. Cultures were transduced with mem-mCherry to evidence the axolemma. Matrix shows tracking of the swellings in time. Shaded area corresponds to the injury stage (from 30 to 120 s, scale bar: 20 µm). **C** Same method as in B applied to the axons transduced with cytosolic-GFP (scale bar: 20 µm). **D** Quantification of the number of swellings in time for each individual axon. mem-mCherry (upper) and cytosolic-GFP (lower) stained axons. Right plot: mean number of swellings per axon (n = 9 for mCherry, n = 4 for GFP). **E** Rasterplot showing the behavior in time of each individual AS for mem-mCherry and cytoslic-GFP. **F** Frequency distribution analysis of swelling thickness and duration. Data are mean ± SEM

For real-time analysis of the axonal morphological changes, we developed an algorithm to track mem-mCherry or cytosolic-GFP transduced axons during the 30 s pre-injury (10 µL/min), 90 s injury (100 µL/min) and 150 s post-injury (10 µL/min) stages (Fig. 2A). The algorithm enables detection of the AS by dividing axons into segments of equal length and measuring the width of each segment. A segment presents a swelling if the thickness of the shaft in that segment is at least 50% larger than the median width of all the segments (Fig. 2B, C; Additional file 5: Video S4, Additional file 6: Video S5).

We observed that AS start to appear in mem-mCherry labelled projections approximately 50 s from the beginning of the injury and throughout the post-injury stage (Fig. 2D). The same behavior was observed when the neurons were transduced with cytosolic-GFP. However, heatmaps of individual axons show that the number of swellings per axon is dynamic in time (Fig. 2D, left). Indeed, some AS are present continuously throughout the measured time (straight horizontal segments), while others are short-lived (dots or short segments) (Fig. 2E). Most of these short-lived swellings can appear for some time and disappear to then reappear again reflecting a waxing and waning behavior. Quantification of the duration of the individual AS showed that majority lasts no longer than 30 s and only a fraction persist throughout the entire post-injury stage (Fig. 2F). The width of the AS ranged between 1.5 and 4 µm, with the most frequent widths of 2.5 µm and 2 µm for mem-mCherry and cytosolic-GFP labelled AS, respectively. In conclusion, using independent markers of axonal membrane and cytosol, we demonstrate that AS that develop in response to the hydrodynamic force, exhibit a waxing and waning behavior, with a minority persisting throughout the post-injury stage.

### Increased axonal calcium levels sustain axonal swellings

**Fig. 3:**
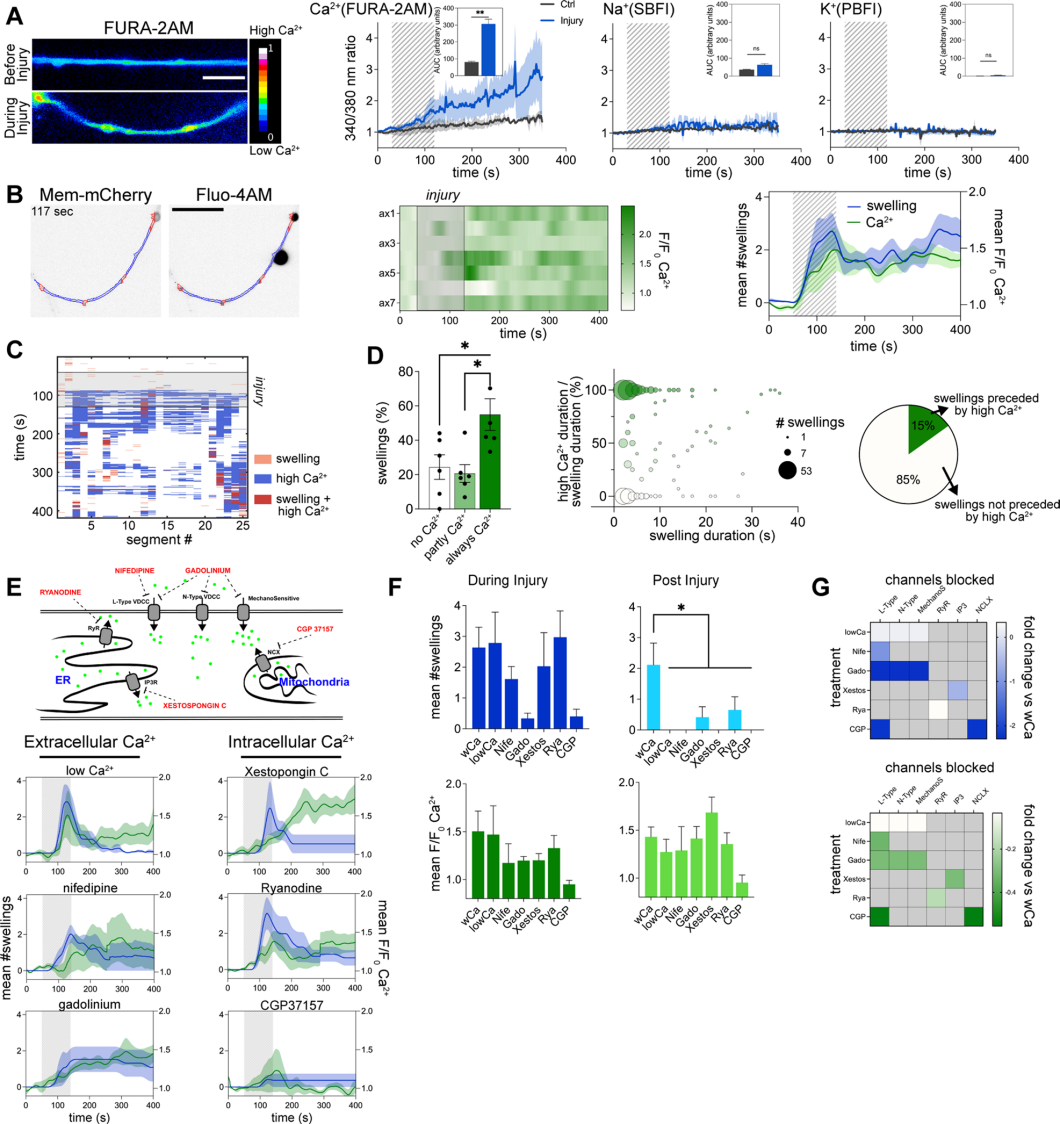
Axonal Ca^2+^ increase is required to sustain swellings. **A** Axonal Ca^2+^, Na^+^ and K^+^ levels during injury measured with the ratiometric probes FURA-2AM, SBFI and PBFI (n = 4–6, scale bar: 10 µm). **B** AS and axonal Ca^2+^ levels during injury. Mem-mCherry transduced neurons were incubated with Ca^2+^ sensor Fluo-4 AM. Middle: mean axonal Ca^2+^ levels during injury. Right: Mean number of swellings and Ca^2+^ levels during injury (n = 7, scale bar: 20 µm). **C** AS and Ca^2+^ tracking across the axonal shaft through the injury. Example of a matrix showing detection of swellings (> 150% width) and high Ca^2+^ (> 1.25x) for each segment of the axonal shaft in time. **D** Percentage of AS per axon that during their duration present always Ca^2+^, part time Ca^2+^ or no Ca^2+^ (left, n = 6, 10–150 swellings/n). Bubble plot shows the relationship between the duration of the AS and the presence of high Ca^2+^ (middle, n = 342 swellings). Pie chart showing the percentage of AS that are preceded (2 s window) by high Ca^2+^ (right, n = 342 swellings). **E** Schematic representation of the compounds used to block different sources of Ca^2+^. Axons were incubated with compounds, subjected to injury and AS formation and Ca^2+^ levels assessed (n = 4–7). **F** Comparison of the effects of different compounds on the mean number of AS and Ca^2+^ intensity levels during and after injury (n = 4–7). **G** Diagram showing the targets of each compound and the mean differences in AS numbers or Ca^2+^ intensity levels compared to the control levels. Data are mean ± SEM (*p < 0.05, **p < 0.01). Statistical comparisons were performed using student t-test (**A**), one-way ANOVA followed by the Tukey’s multiple comparison test (**D**) or Dunnett’s multiple comparison test against the control group (**F**). See also Additional file 7: Fig. S2

Several studies reported changes in different ion concentrations in the axon after injury [24, 27], however, it remains unsettled whether these changes are the result of membrane mechanoporation. To investigate this, we incubated intact axons and axons subjected to injury with fluorophore tagged 3, 10 and 40 kDa large dextrans (Additional file 7: Fig. S2B). No significant differences were found in the uptake of dextrans in intact versus injured axons, suggesting that the applied hydrodynamic force did not produce mechanoporation. We then asked whether injury causes any immediate changes in the axonal ion concentrations. To determine axonal Na^+^, K^+^ and Ca^2+^ concentrations in response to injury, we incubated neurons with membrane permeable SBFI, PBFI and Fura-2 ratiometric probes, and measured the 340/380 nm excitation ratios (Fig. 3A and Additional file 7: Fig. S2A). Measurements showed a significant increase in the axonal Ca^2+^, but not Na^+^ or K^+^ concentrations in response to the hydrodynamic force.

To establish dynamics of the increased axonal Ca^2+^ concentrations, we incubated neuronal cultures with mem-mCherry to visualize AS formation and the Ca^2+^ probe Fluo-4AM to monitor Ca^2+^ levels (Additional file 8: Video S6). We observed a significant surge in the axonal Ca^2+^ concentrations during injury as well as post-injury (Fig. 3B). Since this increase temporally corresponded to the AS formation, we asked if regions of the axons giving rise to AS show specific changes in Ca^2+^ levels. We found that only 54% of the AS displayed increased Ca^2+^ levels during their entire duration, 18% of the AS showed higher Ca^2+^ levels during part of their duration, while 28% showed no Ca^2+^ changes during their lifetime (Fig. 3C, D). There was also no correlation between local Ca^2+^ presence and the AS duration, with only 15% of the AS preceded (2 s prior) by increased Ca^2+^ levels. These results show that increased axonal Ca^2+^ concentrations coincide with the AS formation, but the sites of increased Ca^2+^ levels do not always correspond to the sites of AS formation.

To investigate individual Ca^2+^ sources in the AS formation, we pharmacologically blocked several Ca^2+^ channels or transporters. Extracellular Ca^2+^ was blocked either by incubating axons with nifedipine, gadolinium or by lowering Ca^2+^ in the media, while the intracellular Ca^2+^ sources were blocked using ryanodine, xestospongin C or CGP37157 (Fig. 3E–G and Additional file 7: Fig. S2C). The analysis showed that none of the conditions affected AS formation during the injury, but all of them significantly decreased the number of AS in the post-injury stage. In summary, these results suggest that (1) generation of AS during the injury occurs independently from elevated axonal Ca^2+^ levels, while (2) increased axonal Ca^2+^ levels are required for the maintenance of the AS after the injury.

### Proteomic profile of the axons following injury

**Fig. 4:**
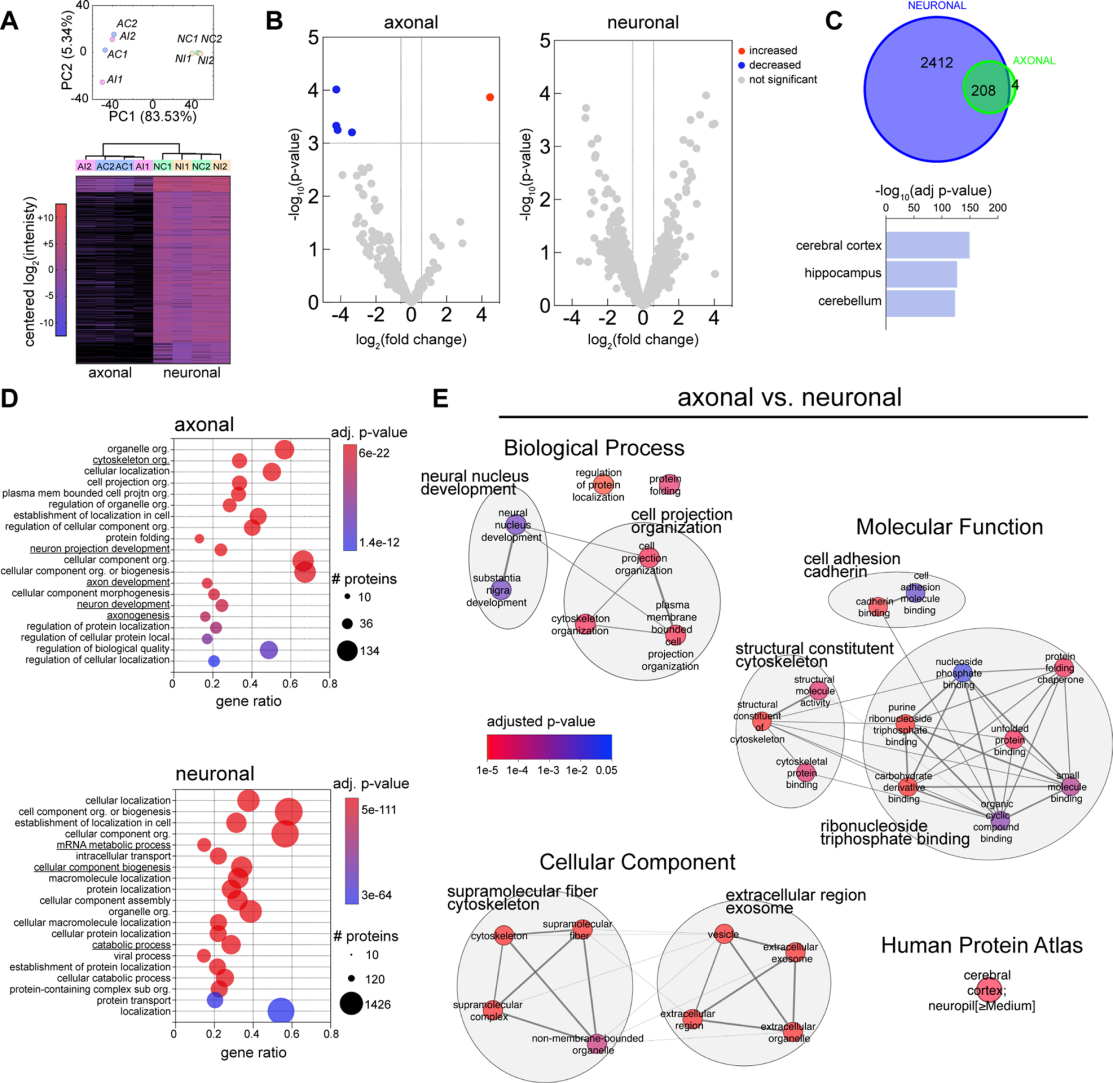
Axonal proteomic profiling before and immediately after injury. **A** Axonal and neuronal compartment fractions (A/N) in both control and injury treatments (C/I) were processed for LFQ mass spectrometry. PCA and heatmap of differentially expressed proteins (n = 2). **B** Volcano plots depicting the quantitative protein changes following injury compared with control treatment for the axonal and the neuronal fractions. **C** Total number of proteins present in the neuronal and axonal fractions (upper panel). Human Protein Atlas top terms of the enrichment analysis for the neuronal fraction proteins (lower panel). **D** Top GO Biological Processes terms for the enrichment of the axonal (upper panel) or the neuronal fraction (lower panel). **E** Enrichment analysis of the axonal fraction proteins using as background the neuronal fraction proteins. Significant terms for each GO category were clustered by similarity and most representative words of the cluster’s terms assigned. See also Additional file 9: Fig. S3

To investigate the mechanisms underlying AS formation, including Ca^2+^-dependent signaling involved in their maintenance, we examined the axonal proteome and phosphoproteome in response to injury. We collected axonal fraction, composed of axons at the site and distal to the injury, and compared it to the neuronal fraction composed of axons, dendrites and somas. Considering remarkably little is known about the axonal proteome, we first performed label free quantitative mass spectrometry with the discovery (Fig. 4 and Additional file 10: Table S1) and then a confirmatory experiment comparing control (intact) versus injured axonal and neuronal fractions (Additional file 9: Fig. S3, Additional file 10: Table S1). The principal component analysis (PCA) and the hierarchically clustered heatmap readily distinguished axonal from the neuronal fraction, however, disclosed no injury induced changes (Fig. 4A, Additional file 9: Fig. S3A and S3B). This was further confirmed by volcano plots comparing the control to the injured axonal and neuronal compartments, which lacked reproducible quantitative protein changes between the discovery and confirmatory experiments (Fig. 4B and Additional file 9: Fig. S3C).

To establish the axonal proteome, we next pooled together control and injured fractions and compared the axonal to the neuronal compartment. The axonal proteome consisted of approximately 10% of the proteins identified in the neuronal fraction. Enrichment analysis showed that the three most significant terms corresponding to the neuronal fraction were related to the central nervous system: cerebral cortex, hippocampus and cerebellum (Fig. 4C and Additional file 9: Fig. S3D). Gene ontology overrepresentation analysis identified several biological processes including axon development and cytoskeleton organization enriched exclusively in the axonal fraction (Fig. 4D). In contrast, mRNA metabolic process, cellular component biogenesis or catabolic process were found exclusively in the neuronal fraction. Enrichment analysis of the proteins in the axonal fraction, using the neuronal fraction as background (Fig. 4E and Additional file 9: Fig. S3E), identified clusters of terms related to cell projection organization, cell adhesion, structural constituent of cytoskeleton, ribonucleoside binding and extracellular exosome (Fig. 4E and Additional file 9: Fig. S3E). We here built an atlas cataloguing the most abundant proteins found in the axons derived from human neurons, which exhibit a proteomic profile reminiscent of the human central nervous system.

### Axonal phosphoproteome following injury

**Fig. 5:**
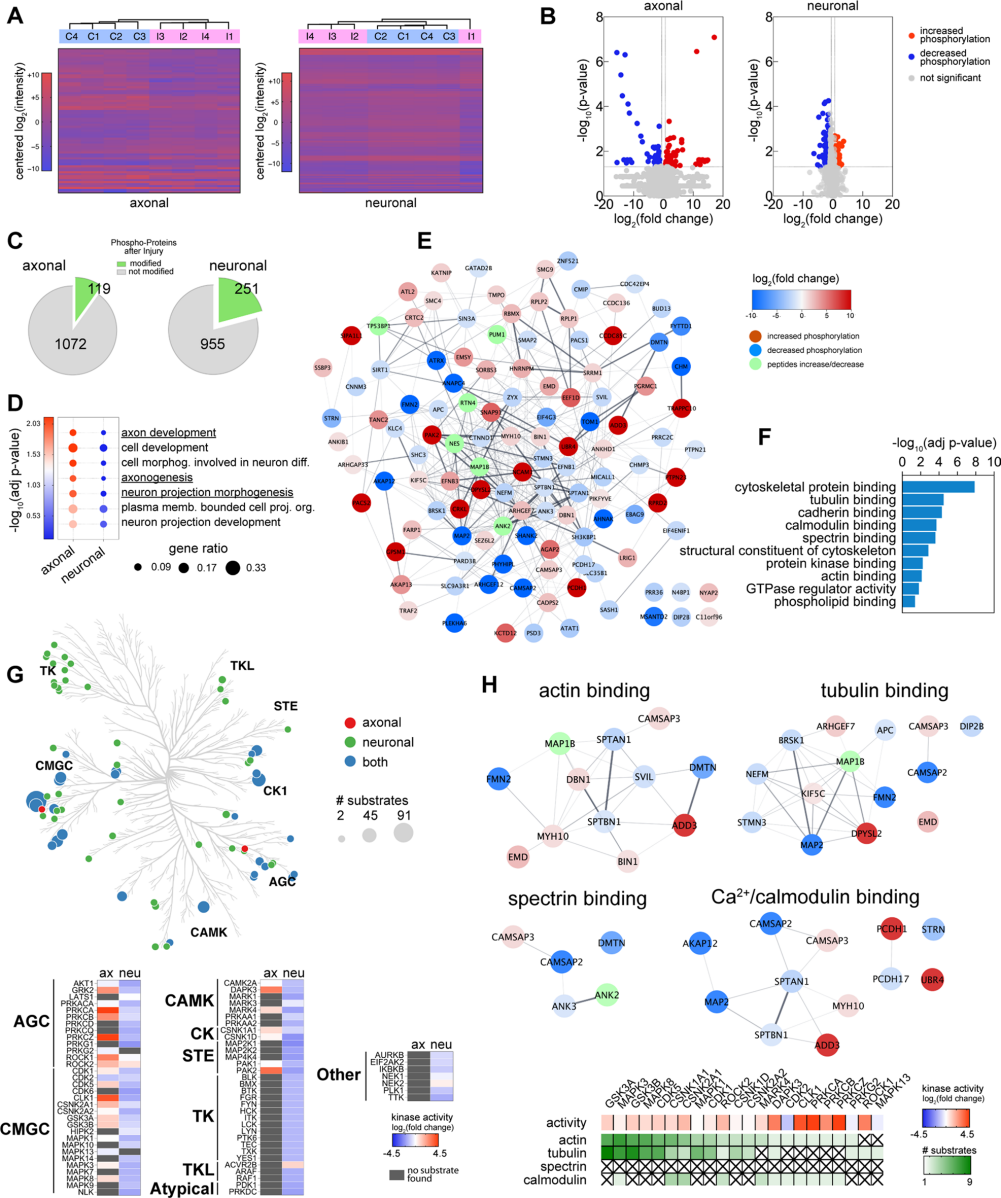
Phosphorylation changes of the axonal proteins following injury. **A** Axonal and neuronal compartment fractions (A/N) in both control and injury treatments (C/I) were enriched for phosphorylation and processed for quantitative mass spectrometry. Heatmaps of phospho-peptides levels comparing injury versus control treatment for both axonal and neuronal fractions (n = 4). **B** Volcano plots of phosphopeptides levels comparing injury versus control treatment for both the axonal and neuronal fractions. **C** Pie charts depict the number of identified proteins that significantly increase or decrease their phosphorylation and the number of proteins that are not modified after injury. **D** Enrichment analysis (GO Biological Processes) of the proteins that are significantly regulated by phosphorylation after injury, using as background all the detected phosphoproteins. Comparison of significant terms between the axonal and neuronal fractions. **E** Stringplot showing all the phosphoproteins in the axonal fraction significantly changed after injury. **F** Overrepresentation analysis of the proteins in (**E**) showing top significant terms for GO molecular functions. **G** Kinome tree highlighting predicted kinases in both fractions, based on the significantly changed phosphopeptides as substrates (left). List of kinases and their families with the predicted activity based on their substrate’s fold change. **H** Stringplots of phosphoproteins present in specific cytoskeletal terms found in (**F**) and their kinases predicted activity

Protein phosphorylation is the major mechanism through which protein function is regulated in response to stimuli. To investigate the immediate response of axonal proteins to injury, we enriched samples for phosphopeptides and examined their phosphorylation status using mass spectrometry (Fig. 5 and Additional file 11: Table S2). In the axonal and neuronal fractions, respectively, we identified 78 and 80 differentially up-regulated phosphopeptides following injury, while 73 and 292 phosphopeptides were down-regulated (Fig. 5A, B). We estimated that out of a total of 3345 and 3444 phosphopeptides corresponding to 1191 and 1206 proteins in the axonal and neuronal fractions, approximately 10% of the axonal and 21% of the neuronal fraction proteins underwent changes in their phosphorylation status in response to injury (Fig. 5C). Enrichment analysis of the proteins that changed their phosphorylation status after injury versus all the identified phospho-proteins showed that pathways related to the axon development are overrepresented in the axonal fraction, while no significant enrichment was found in the neuronal fraction (Fig. 5D).

We then performed a detailed analysis of all the protein’s phosphorylation sites and their previously described functions (Fig. 5E and Additional file 11: Table S2). We identified phosphorylation changes in protein sites previously reported to regulate protein function and cytoskeletal organization in DMTN (S92), MYH10 (S1952), SLC9A3R1 (S280) and STMN3 (S60) [36–39]. An enrichment analysis of Molecular Function showed that the set of proteins regulated by phosphorylation in the axon immediately after injury is overrepresented in functions such as cytoskeletal protein binding (including actin, spectrin and tubulin), binding to calmodulin and protein kinases, and regulators of GTPase activity (Fig. 5F). Interestingly, no terms related to stress or degeneration were significantly enriched.

### Axonal kinome following injury

Identification of protein kinase binding among the most significantly changed molecular functions prompted us to predict putative kinases involved in the phosphoprotein changes triggered by injury (Fig. 5G and Additional file 12: Table S3). The predicted axonal kinome resulting from injury revealed that the most represented families of kinases were the CMGC (CDK, MAPK, GSK and CLK), AGC (PKA, PKG and PKC) and CAMK. The majority of the kinases found in the axonal fraction are predicted to be upregulated, with stronger activities present in PRKCA/Z/B, CLK1, PAK2, DAPK3, ROCK1/2 and GRK2. In contrast, the neuronal fraction showed a general downregulation of activity, presenting also members of the TK and TKL families of kinases.

We last linked axonal actin, tubulin, spectrin and calmodulin binding networks with their predicted kinases into discrete functional clusters (Fig. 5H). We found changes in proteins participating in actin bundle stabilization or assembly (DMTN, FMN2) and linkage of actin to membrane (SVIL), proteins that have microtubule destabilizing activity (STMN3) or acetylation of tubulin (DIP2B), and proteins that participate in the localization of channels in the axolemma (ANK2 and 3). Among the calmodulin regulated proteins, CAMSAP2 and 3 bind the minus-end of microtubules and spectrin, SPTBN1 and SPTAN1 are part of the spectrin-actin subaxolemmal periodic cytoskeleton, ADD3 caps actin filaments and promotes the formation of the spectrin-actin network. In terms of kinases, we predicted their activity and the number of targeted substrates. We identified PRKCA/B/Z, ROCK1, CLK1 and DAPK3 as the overall most activated kinases. MAPK3 (ERK1), MAPK8 (JNK1), GSK3A/3B and CDK5, on the other hand, were found to target most substrates. There were no predicted kinases within the spectrin binding network.

### Reorganization of the axonal cytoskeleton following injury

**Fig. 6:**
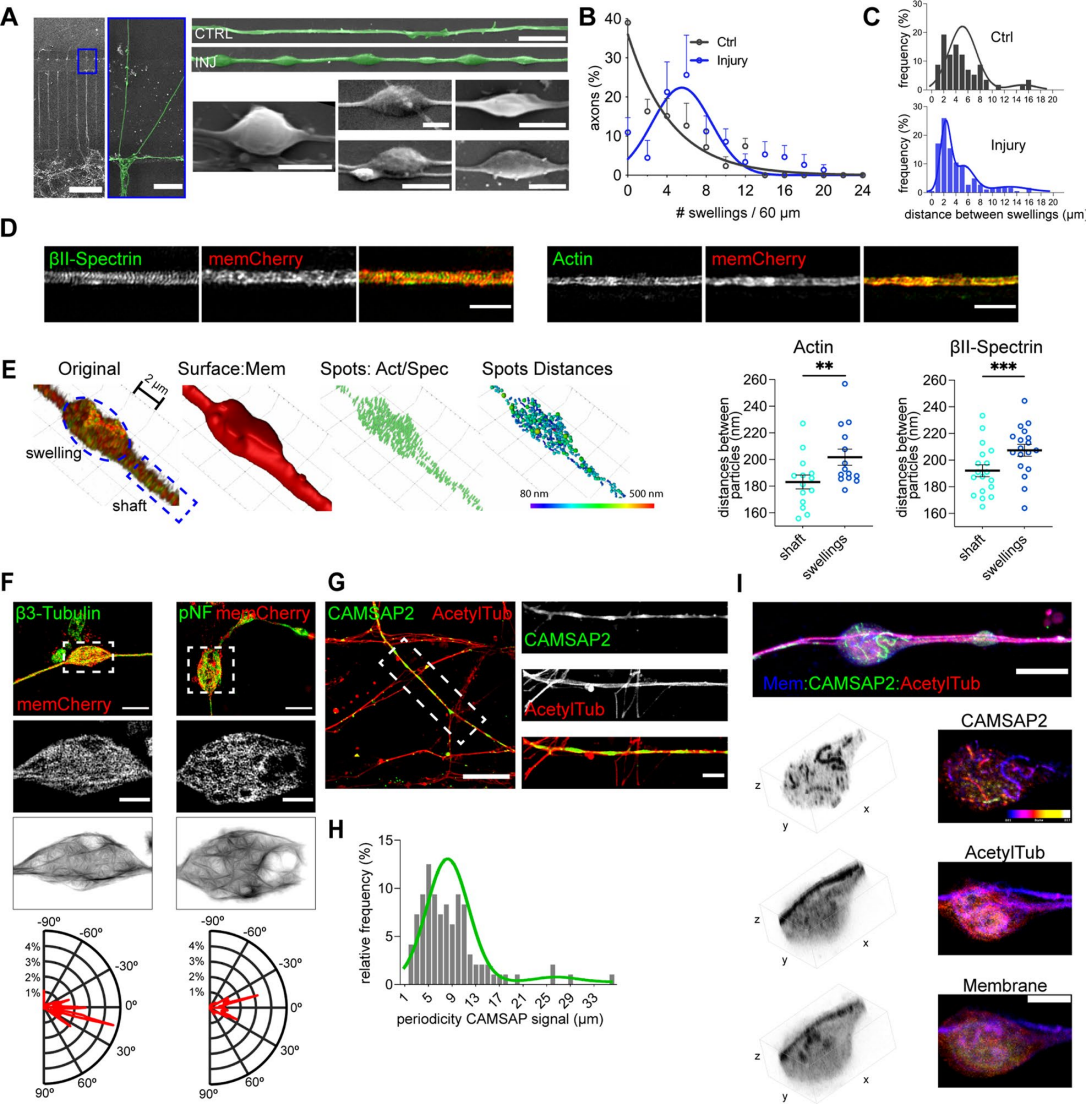
Structural reorganization of the axonal cytoskeleton following injury. **A** Representative scanning electron microscopy images of AS in control and axons subjected to injury (scale bar chamber: 200 µm, close-up: 20 µm, right horizontal: 5 µm, swellings: 2 µm). **B** Frequency distribution of the number of AS measured per axon (n = 5). **C** Frequency distribution of inter-swelling distances for control and injured axons. The distribution was fitted with three Gaussian curves with means of 3.09 µm, 6.12 µm and 13.87 µm (variances: 0.75, 3.02, 8,76 µm, respectively, control n = 57, injury n = 181). **D** SIM images of axons transduced with mem-mCherry and immunostained for βII-Spectrin or actin (scale bar: 2 µm). **E** Identification of AS and shaft ROIs, masking for surface (mem-mCherry), identification of actin or βII-Spectrin particles and measurements of the distances between the closest neighbours. Distances in shaft vs distances in swellings for actin and βII-Spectrin (n > 14). **F** SIM images of axons transduced with mem-mCherry and immunostained for βIII-tubulin or pNF. Images were processed and magnitude of directionality measured and plotted in polar plots (scale bar upper: 5 µm, middle: 2 µm). **G** Representative image of the axons immunostained for CAMSAP2 and acetylated tubulin after injury (scale bar: 20 µm, close-up: 5 µm). **H** Frequency distribution of the periodicity of CAMSAP2 staining along the axons after injury (n = 96). **I** Representative image (z-projection) of an axon transduced with mem-mCherry and immunostained for CAMSAP2 and acetylated tubulin (scale bar: 10 µm, close-up: 5 µm). Data show mean ± SEM (**p < 0.01, ***p < 0.001). Statistical comparisons were performed using the student t-test (**E**). See also Additional file 13: Fig. S4

Most of the phosphorylation changes of the axonal proteins following injury indicate cytoskeletal regulation. This led us to investigate the morphology and structure of the AS. We first compared morphology of the AS in control versus injured axons using scanning electron microscopy (Fig. 6A, B). We found significant increase in the number of AS in the injured compared with control axons. These were most commonly observed as strings of up to 6 equally spaced AS with shorter inter-swelling distances. Although larger AS were occasionally observed, the majority of AS forming immediately following injury was indistinguishable in shape and size from those found in control axons (Additional file 13: Fig. S4A).

We next examined cytoskeletal structures within AS. We transduced axons with mem-mCherry, stained for actin or βII-spectrin and then imaged axons using Structured Illumination Microscopy (Fig. 6D). To calculate the distance between particles, we measured the closest neighbour distance for all the particles detected inside the axon (Fig. 6E and Additional file 13: Fig. S4B). Analysis in the axonal shaft adjacent to the AS revealed 183.1 ± 5.22 nm actin and 192.1 ± 4.43 nm βII-spectrin periodicities consistent with previous reports [40]. In contrast, actin and βII-spectrin periodicities within the AS amounted to 201.8 ± 6.1 nm and 207.4 ± 4.46 nm, respectively (Fig. 6E, Additional file 13: Fig. S4C and S4D). Increased distances between actin and βII-spectrin particles suggest stretching of the actin-spectrin rings in the AS. Measurements of directionality of microtubule or neurofilament tracks by polar plots showed either a linear or a criss-cross pattern within the AS (Fig. 6F).

Stretched actin-spectrin rings and reorganized microtubules support a role of cytoskeletal regulatory proteins in the AS formation. To test this, we investigated CAMSAP2, which showed decreased phosphorylation after injury and was previously reported to interact with both spectrin and microtubules. Imaging revealed discrete stetches of CAMSAP2 immunoreactivity along the axons (Fig. 6G). These linear stretches of CAMSAP2 adopted a snake-like shape in a subset of AS (Fig. 6I). To investigate this change further, we performed real-time imaging of CAMSAP2-GFP and found bending of CAMSAP2 stretches during injury which acquired and maintained the snake-like shape after injury (Additional file 13: Fig. S4E and Additional file 14: Video S7). These findings suggest involvement of CAMSAP2 in the microtubule reorganization within the AS, which is further supported by the periodical accumulation of CAMSAP2, reminiscent of the AS distribution along the axons (Fig. 6H). These experiments demonstrate reorganization rather than disruption of the axonal cytoskeleton within the AS and support a role of cytoskeletal regulatory proteins in the AS formation.

### Adaptation of the axonal transport in response to injury

**Fig. 7:**
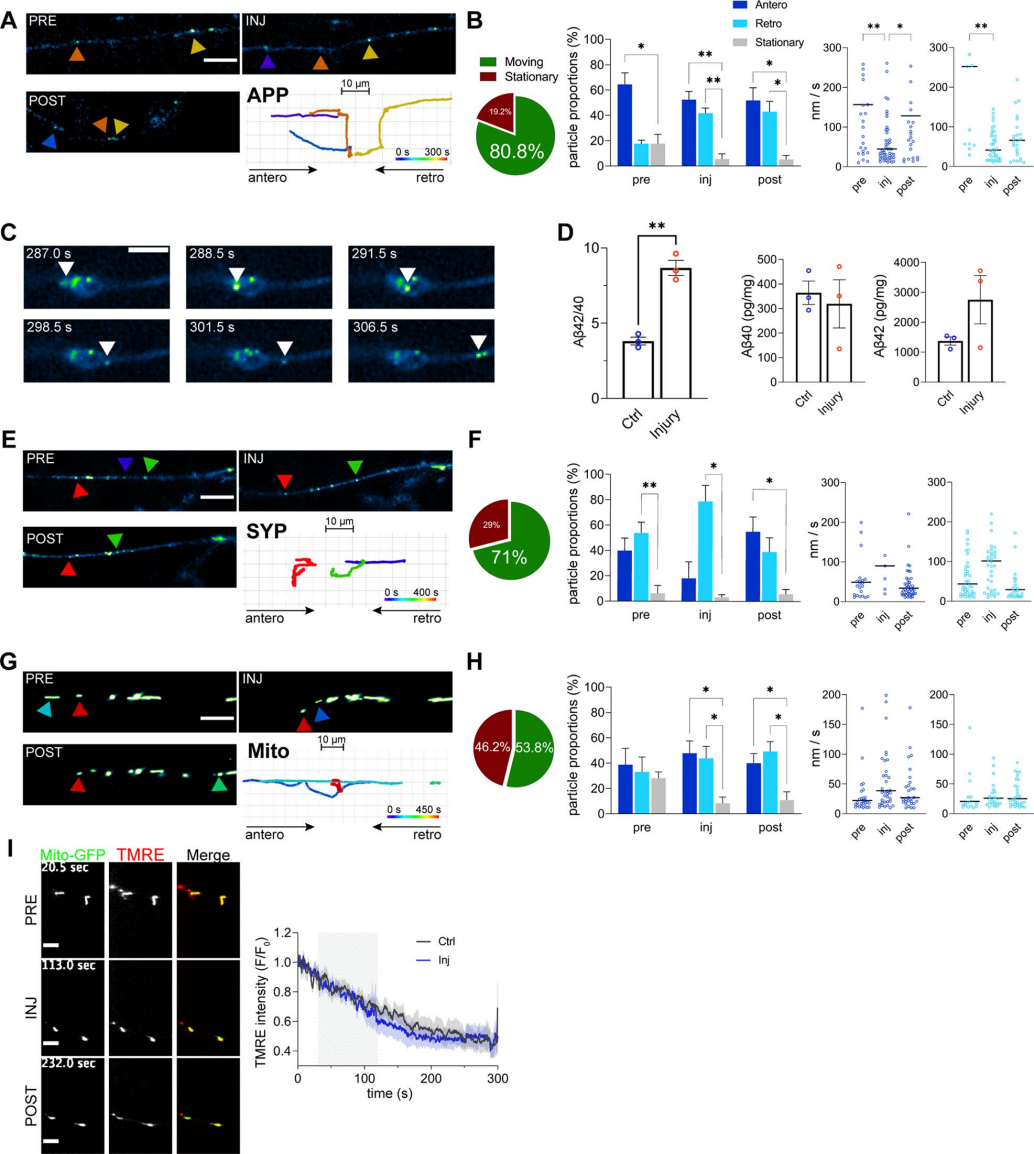
Adaptation of the axonal transport in response to the injury. **A** and **B** Tracking of APP-GFP particles in pre, during and post-injury stages (A, scale bar: 10 µm). Proportion of moving and stationary particles in the full tracking time (pre, injury and post injury stages) (**B**). Anterograde, retrograde and stationary movement particle proportions for each stage (n = 4). Anterograde and retrograde median velocities in the three different stages (n = 33–56). **C** Sequence of an APP-GFP particle moving anterogradely through an axonal swelling (scale bar: 5 µm). **D** ELISA Aβ40 and Aβ42 quantification in the axonal compartment comparing control and injured axons (n = 3). **E** and **F** Tracking of Synaptophysin-GFP particles in pre, during and post-injury stages (**E**, scale bar: 10 µm). Proportion of moving and stationary particles in the full tracking time (**F**). Anterograde, retrograde and stationary movement particle proportions for each stage (n = 4). Anterograde and retrograde median velocities in the three different stages (n = 13–40). **G** and **H** Tracking of Mito-GFP labeled mitochondria in pre, during and post-injury stages (**G**, scale bar: 10 µm). Proportion of moving and stationary particles in the full tracking time (**H**). Anterograde, retrograde and stationary movement particle proportions for each stage (n = 5). Anterograde and retrograde median velocities in the three different stages (n = 21–40). **I** Mitochondrial membrane potential levels during injury measured with TMRE probe (n = 7). Data are mean ± SEM (*p < 0.05, **p < 0.01). Statistical comparisons were performed using student t-test (**D**), two-way ANOVA followed by Dunnett’s multiple comparison test against stationary group (proportions: **B**, **F** and **H**) or Kruskal–Wallis test followed by Dunn’s multiple comparisons test against pre-injury (speed: **B**, **F** and **H**)

If the cytoskeleton is reorganized rather than disorganized immediately following injury, then axonal transport and function should not be disrupted. To test this hypothesis, we assessed microtubule dependent transport of two well-established axonal cargos transported by different molecular motors, APP-GFP or synaptophysin-GFP, and of an organelle, mito-GFP. We acquired movies of axonal transport prior to, during and after axonal injury, and developed a method to track particle movement in bending axons during the injury (Fig. 7).

APP-GFP particles demonstrated increased retrograde axonal transport at the expense of reduced number of stationary particles during and following injury. This was accompanied by decreased anterograde and retrograde average velocities during injury (Fig. 7A, B; Additional file 15: Video S8). Axonal transport of the APP particles occurred without interruptions also within AS forming during injury (Fig. 7C and Additional file 16: Video S9). Since post-mortem work indicated perturbed proteolytic processing of the APP during axonal injury [41], we next tested APP processing and found significantly increased Aβ42/40 ratio in the axonal fraction (Fig. 7D). Conversely, synaptophysin particles showed no significant changes in the directionality during injury, however, they did exhibit significant increase in anterograde and decreased retrograde movement following injury (Fig. 7E, F; Additional file 17: Video S10). This was not accompanied by changes in velocities.

We last examined axonal transport of mito-GFP including mitochondrial labelling with a membrane potential sensor, the tetramethylrhodamine ethyl ester perchlorate (TMRE). During and following injury, we recorded significant increase of both anterograde and retrograde mitochondrial transport, while no changes were detected in average velocities (Fig. 7G, H; Additional file 18: Video S11). Mitochondrial membrane potentials also showed no differences in the normalized TMRE intensities of mitochondria prior to, during and following axonal injury (Fig. 7I; Additional file 19: Video S12). These functional assessments of the axons indicate that axonal transport and function do not undergo any major impairments during and following axonal injury.

## Discussion

This study dissects events and mechanisms during the earliest axonal response to physical injury. We found a rapid appearance of AS as a result of the mechanical stress. AS were characterized by an increased spacing of the actin-spectrin rings and a conformational reorganization of the microtubule and neurofilaments tracks, concomitant to phosphorylation changes of cytoskeletal regulators. AS became stable and endure in time only if sustained by high axonal Ca^2+^ concentrations. Finally, we observed that axonal transport and function are preserved throughout and following injury, revealing that initially AS maintain a degree of functionality.

### Axonal injury system

Our model has unique features including: (1) exclusively axonal changes can be followed during and after injury in real-time, (2) axonal material subjected to injury can be isolated for biochemical, transcriptomic or proteomic studies, and (3) the use of human derived mature and functional neurons makes the system a better option for clinically relevant studies of axonal injury. In addition, our system has properties that make it comparable to other models, such as the applied force and the size of the AS. We set the duration of the injury to 90 s considering fluorescent beads require this time to reach their maximum speed. To establish the optimal stress, we examined axonal response to different pump flow rates and selected the flow rate at which we observed maximum AS with minimum axotomy (100 µL/min). Both experimentally and by finite element analysis, we found that under these injury parameters, the maximum stress applied to the axons equals 40 Pa. This stress is in the range of the ones used by others in comparable in vitro models [26, 42, 43]. The translation of the in vitro to the in vivo models in terms of applied stress is hindered by the complexity and heterogeneity of brain’s cellular and extracellular components. However, in terms of the consequences of the mechanical stress on the axons, the AS sizes that we report both by live imaging of the axons and by SEM are strikingly similar to the previously reported sizes in vivo. Indeed, the Thy1-YFP mice form AS that are 2–3 µm in length and 2 µm in diameter 15 min following fluid percussion induced injury [44].

### Mechanism of AS formation

In light of our experiments, we can distinguish two consecutive stages of the generation of AS. A first stage, characterized by the initial formation of AS and a second stage that involves the sustenance of the AS following injury. Both stages are normally accompanied by increased axonal Ca^2+^ levels, which is in agreement with previous reports [24, 25]. However, our pharmacological experiments demonstrated that the role of Ca^2+^ becomes critical for the perpetuation of the AS, while the formation of AS during the injury does not depend entirely on Ca^2+^. Initial formation of AS could be explained as the consequence of fast and reversible mechanisms, that can be the result of either osmotic changes inside the axon [45] or of a contractility or relaxation mechanism derived from MYH9 or MYH10 interaction with the axonal cytoskeleton [46, 47]. In addition, we identified phosphorylation changes of formins (FMN2), myosin (MYH10) and predicted an upregulation of the Rho/ROCK pathway. These are previously described components of the contractile actin cortex which participates in plasma membrane blebbing mechanism [48, 49], and can explain a relatively fast, reversible and Ca^2+^ independent AS formation.

The requirement of high Ca^2+^ levels to sustain AS in time suggests that Ca^2+^ underlies stable changes like the rearrangement of the axonal cytoskeletal components, which may not be promptly reversible. Phosphoproteomics predicted an upregulation of Ca^2+^ dependent kinases immediately following injury that were previously reported to regulate cytoskeletal dynamics, namely PRKCA/B [50], CAMK1 [51], 2A and 4. Besides, we observed upregulation in JNK1, CDK5, GSK3A/B and ROCK1/2, all which have been previously described to participate in axonal homeostasis [52–54]. Indeed, through phosphoproteomics we found that the main groups of proteins subjected to regulation via phosphorylation after injury are components of the cytoskeleton (e.g., ANK2, ANK3, ADD3, NEFM, SPTBN1, SPTAN1) or participate in cytoskeleton regulation (e.g., DMTN, SVIL, FMN2, STMN3, DIP2B). Our analysis of cytoskeletal changes shows that the spacing of actin-spectrin rings is increased in the AS, but not in the adjacent shaft, which suggests that actin and spectrin rings participate in the enlargement of the AS membrane. It has been recently shown that actin and myosin work together to maintain the axonal diameter [46, 47], and that α-adducin cap regulates the diameter of the axons [55]. In line with these studies, our experiments found phosphorylation changes in several actin-associated proteins, including ADD3 and MYH10. Moreover, microtubules and neurofilaments showed no signs of disintegration in AS, but a clear spatial reorganization supporting the transport of cargos along the axon. Several proteins that participate in microtubule binding (e.g., STMN3, MAP1B, DYPSL2, DIP2B, BRSK1 or CAMSAP) were also found to be differentially phosphorylated, strongly suggesting an active process of cytoskeletal remodelling as a result of the injury. Changes in the axonal cytoskeleton following injury have been observed in several in vitro models. Among cytoskeletal changes within the AS, studies report interrupted microtubule tracks [19] as well as increased immunoreactivity of neurofilaments [32], F-actin and Tau [15, 19]. In case of extensive breakage and disintegration of microtubules, the axonal transport is expected to be completely interrupted. Live imaging recordings of the axonal transport immediately following injury in the present as well as in the previous studies, however, show only mildly impaired axonal transport [26, 56]. Transport analyses of different cargos at later time points are needed to understand the long-term changes in transport and its relationship with AS. Moreover, studies in mouse models (e.g., Thy1-YFP mice) that test and modulate the rapid phosphorylation of cytoskeletal components identified in the present work, can help in the development of therapeutic strategies to minimize the impact of traumatic injury on the axonal homeostasis.

After injury we observed a periodic distribution of the AS along the shaft. This suggests that proteins along the axon involved in some particular process determine the location where AS develop. Previous studies have reported specific Ca^2+^ increases in regions of the axons where AS develop. These regions were characterized by higher abundance of NCX1 and N-type VGCC [23] or TRPV4 [26] channels, which allow Ca^2+^ entry under appropriate conditions. In contrast, our results show that focal elevated Ca^2+^ levels do not necessarily determine the presence of AS. To explain the location where the AS develops, we found a candidate in CAMSAP. This protein presents a stretch-like disposition along the axons, exhibits microtubule minus-end stabilizing properties and also binds spectrin as well as calmodulin. By immunostaining, we observed that CAMSAP2 presents a periodic distribution after injury, reminiscent to the swelling distribution. Furthermore, it was usually present in the AS following a snake-like disposition and interestingly, phosphorylation changes in CAMSAP were found also after injury. Further studies are needed to understand whether CAMSAP determines the location of the AS in the axonal shaft through microtubule and/or cortical cytoskeleton changes.

### AS and pathology

The present work focused largely on the real-time examination of the immediate response of the axons to the injury and showed that in the earliest stages of the axonal injury, AS represent an adaptation rather than an impairment of their structure and function. This concept is also postulated in recent studies where it was shown that in Purkinje cells AS develop as a physiological response in normal development, or compensatory response in disease [57, 58]. Also, as a consequence of TBI, AS develop in myelinated axons 3 days after injury, correlating with demyelination and impaired speed of signal conduction [59]. These parameters, however, are partially restored 2 weeks after TBI. In addition, some effects that resulted from injury in other studies, were not present in our model. Among them, mitochondrial membrane potential remains unchanged during the injury and mitochondrial transport is not disrupted. There was a lack of significant membrane mechanoporation [43], and we didn’t observe an intra-axonal increase in Na^+^ or K^+^ levels during injury [60]. Finally, regulation of kinases or proteins involved in signaling of cell death or neurodegenerative pathways was not present.

It’s possible that any of the cytoskeletal, phosphorylation or transport changes that we observed are the starting point that can evolve and acquire pathological characteristics. Even though the cytoskeletal rearrangements are functional in terms of axonal transport, they can be deleterious to the axonal homeostasis if they are not reverted in the long run. For example, we didn’t observe APP accumulation during or immediately following injury, but we observed an increase in the intra-axonal Aβ42/Aβ40 ratio. Given the complex relationship between transport and APP processing, this could be the first step to aberrant Aβ accumulation as observed in a stretch in vitro model or in the post-mortem AS of individuals with TBI [56, 61]. Also, immediately after injury, we observed phosphorylation changes in MAP1B (S1396 and S1400) and in NEFM (S837) in the axonal fraction. Interestingly, these sites were previously reported to be specifically regulated in phosphoproteomics analysis of Alzheimer’s disease brains [62, 63]. On the long run, these phosphorylation changes in fundamental proteins of the axonal cytoskeleton could result in impaired axonal homeostasis. Exploration of this mechanism may lead to understanding of the contribution of TBI as a risk factor for later development of AD [64]. Last but not least, it can well be that repetitive or chronic injury is required to produce pathological AS. If this is the case, all the observed changes in cytoskeleton and transport should, through time, be reverted to normal.

Although further studies are needed to decipher the mechanisms of transition between physiology and pathology of the AS, the observed structural reorganization with preserved function of AS following injury offers a window of opportunity for prevention and treatment of brain trauma and other axonal pathologies. Altogether, our findings lay down a molecular, structural and functional framework necessary to build a comprehensive mechanistic understanding of the axonal response to injury from the earliest moments to the post-injury period.

## Materials and methods

### Cell lines

The Human Neural Stem Cells (NSCs) were obtained from GIBCO. They are derived from the NIH approved H9 (WA09) human embryonic stem cell line. They retain their normal female human karyotype and the potential to differentiate into neurons and glial cell populations following multiple passages.

NSCs were maintained and amplified up to 4 passages using the NSCs media (KO DMEM/F12; StemPro Neural Supplement 2%, Glutamax 1%, bFGF 20 ng/mL, EGF 20 ng/mL) in matrigel coated plates.

### Neuronal differentiation

The human NSCs (Gibco) (passage < 5) were seeded in matrigel coated p100 Petri dishes, maintained in NSC culture media (KO DMEM/F12, StemPro Neural Supplement 2%, Glutamax 1%, bFGF 20 ng/mL, EGF 20 ng/mL) and changed every other day until confluence. After confluence, media was changed to Neuro Progenitor induction Media (NPM) (DMEM/F12, B27 1%, N2 0.5%, Glutamax 1%) for 7 days, changing the media completely every other day. Then cells were washed once with PBS, detached with accutase (5–10 min), resuspended in DMEM and centrifuged at 250 g. Supernatant was discarded and cells resuspended in Neuronal Optimized Media complete (NOMc) (DMEM/F12, B27 2%, N2 1%, laminin 1 µg/mL, cAMP 100 nM, ascorbic acid 200 ng/mL, BDNF 10 ng/mL, GDNF 10 ng/mL, IGF 10 ng/mL). Depending on the experiment, different densities of progenitors were seeded in matrigel coated surface (DIV0) and maintained in NOMc for neuronal maturation. Unless stated, experiments were performed at DIV40 of neuronal maturation.

### Chamber assembly and neuronal growth

The microfluidic chambers (Xona microfluidics) were cleaned with ethanol and the coverslips coated overnight with a poly-ornithine solution (0.1 mg/mL in PBS). Before bonding, poly-ornithine from coverslips and ethanol from chambers were both removed by thorough washes with distilled water. Bonding was performed by placing the microfluidic device over the dry coverslip and applying a uniform pressure on the upper surface of the microfluidic chamber as detailed in the manufacturer’s instructions (Xona microfluidics). After assembling, the reservoirs of the microfluidic chamber were filled with PBS, avoiding to generate any bubble in the channel system. PBS was removed (not completely to avoid bubbles) and matrigel added in all of the reservoirs to completely coat both compartments. Chambers were maintained in the incubator at 37 °C for at least 1 h. Immediately before seeding, matrigel was removed from the chambers and NPCs (cf. Neuronal differentiation) seeded in the top well of the somatic compartment (300,000 cells in 50 µL of NOMc). Cells flowed from the top well through the somatic compartment where they attached. Chambers were then placed into the incubator for 30 min, after which the wells were topped up with NOMc. Media levels in the wells of the somatic compartment should be kept higher than the levels of the axonal compartment. Cultures were maintained by changing media completely once per week and equilibrated three days after. After approximately 20 days in culture, we observed axons starting to cross the flow channel.

### Injury protocol

The syringe pump (NE-1002X, New Era Pump Systems Inc) was fitted with a syringe (HSW, Norm-Ject 12 mL) and connected to a tubing (GE Healthcare peek tubing green 0.75 mm i.d., 1/16 o.d.; #18-1112-53) entering the microscope plexiglass incubator. The syringe and the tubing were loaded with PBS and purged in a way that liquid continuity was not interrupted by any bubble. Microfluidic chambers were placed in the confocal microscope stage (Zeiss Confocal LSM780, Zeiss Live LSM7) preheated to 37 °C and equilibrated to 5% CO_2_. Depending on the experiment, medium in the microfluidic chamber was completely replaced by aCSF (NaCl 121 mM, KCl 2.5 mM, CaCl2 2.2 mM, MgSO4 1 mM, NaHCO3 29 mM, NaH2PO4 0.45 mM, Na2HPO4 0.5 mM, glucose 20 mM, pH = 7.4) prior to the experiment. The tubing from the syringe pump was inserted into the opening of the flow channel of the microfluidic chamber and a basal flow in the withdrawal direction (1–10 µL/min) maintained until the injury was performed. For the injury, a 100 µL/min flow rate was applied for 90 s in the withdrawal direction, after which it was immediately reversed for 10 s and returned to the basal flow for the time required by the experiment.

### Speed measurements and analysis

3–3.4 µm fluorescent beads (BioLegend) were resuspended in PBS and 80 µL of the solution loaded to the middle bottom well. All the other wells were topped up with PBS. The system was mounted in the microscope stage and starting from basal flow of 10 µL/min, different pump flow rates (30, 50, 100, 150 and 200 µL/min) were selected in the syringe pump. Using brightfield and epifluorescence illumination, movies were recorded (@800 fps) in the flow channel for 90 s. In each movie the speed of 10 beads was measured by kymograph plots every 10 s interval. The continuity equation was used to estimate the speed in the flow channel:

$$A_{sy} \times V_{sy} = A_{ch} \times V_{ch} .$$

where A: area, V: velocity, sy: syringe, ch: channel.

A simplified version of the microfluidic chamber geometry has been analyzed using commercial Finite Elements Analysis software suite (Comsol Multiphysics 5.2) to calculate velocity profile and shear stress at the walls.

### Force measurements and analysis

For measuring the stress at different pump flow rates, we used a urethane microstamp matrix printed with round pillars of 5 µm diameter: 10 µm tall: 12 µm spacing (RMS microstamps). The microstamp was embedded in OCT and cut in a cryostat into 40 µm sections. With the help of a scalpel and a stereomicroscope, the resulting sections were further cut to obtain pieces of approximately 50 µm in length, 30 µm in height (including pillars height 10 µm) and 40 µm in depth. One piece per microfluidic chamber was placed in a way that the pillars were facing to the side (perpendicular to the flow channel long axis). In this position, when placed in the microscope, the pillars could be seen from the side, making it easier to visualize the bending. Microfluidic chambers were bonded to the coverslip and filled with PBS. The chamber was connected to the syringe pump and placed in the stage of the microscope. A basal flow rate of 10 µL/min was maintained before applying a 400 µL/min flow rate for 90 s. Movies were recorded and bending of the windward pillar measured. Maximal force acting on the pillar was calculated based on Hooke’s law [65]:

$$F = \frac{{3 \cdot \pi \cdot E_{u} \cdot D^{4} }}{{64 \cdot L^{3} }} \cdot \delta$$

where F is the force applied to the pillar, Eu is the Young’s modulus of urethane (1.38 MPa), D is the diameter of the pillar (5 µm), L is the length of the pillar (10 µm) and δ is the displacement of the center of the pillar.

For further calculating the stress of the axon we used:

$$\upsigma = {\text{F/A}}$$

where σ is stress, F is force, and A is area of the axon (typical axon of length 60 µm and 1 µm diameter).

### RT-PCR

Total RNA was isolated from cultures at various stages of differentiation (NSC, NPC and mature neurons) using the RNeasy Plus Micro kit (Qiagen) according to the manufacturer’s protocol. Samples for each stage were taken for NSCs stage at confluence, for NPC stage at the end of the progenitors differentiation (7 days in NPM media) and neuronal stage at DIV40 of the neuronal maturation (cfr. Neuronal differentiation). HEK cells were used as the control. Reverse transcription was performed with the Transcriptor First Strand cDNA Synthesis Kit (Roche) in a two-step RT-PCR. Briefly, 250 ng of RNA were mixed with 1 μL of Anchored-oligo (dT)_18_ primer and water to a final volume of 13 μL. The denaturation step was performed at 65 °C for 10 min. Samples were then cooled on ice and 7 μL of RT mix were added. RT conditions were: 10 min at 25 °C, 30 min at 48 °C, and 5 min at 95 °C. For the qPCR, 1 μL of the 10 × cDNA dilution was used. qPCR reactions were performed in triplicate with 1 μL of cDNA and 9 µL of Power SYBR® Green PCR Master Mix (Applied Biosystem) in a final volume of 10 µL using the LightCycler® 480 Instrument II (Roche) under the following cycling conditions: initial denaturation at 95 °C for 3 min, 50 cycles of 30 s at 95 °C, 30 s at 58 °C and 45 s at 72 °C, and a final step of 1 min at 72 °C. Data were analysed to obtain the ΔCT per target gene. Data from five independent experiments were pooled and normalised against the three housekeeping genes (RPL13, RPL27 and β2M). The sequences of the primer pairs for the specific target genes were:

**Table:** 

genes	Fwd (5ʹ–3ʹ)	Rev (5ʹ–3ʹ)
β2M	CTCGCGCTACTCTCTCTTTCTG	GCTTACATGTCTCGATCCCACT
RPL13	GGACCTCTGTGTATTTGTCAATTTT	GCTGGAAGTACCAGGCAGTG
RPL27	ACATTGATGATGGCACCTCAG	CCAAGGGGATATCCACAGAGT
GFAP	AGGAAGATTGAGTCGCTGGAG	CGGTGAGGTCTGGCTTGG
MAP2	ATTCCGAGGTTCCAACACAC	ACCAGCCATTGAAGAAATGC
OLIG2	CGGCTTTCCTCTATTTTGGTT	GTTACACGGCAGACGCTACA
PAX6	CAGGTGTCCAACGATGTG	GTCGCTACTCTCGGTTTACTAC
SOX2	GCACATGAACGGCTGGAGCAACG	TGCTGCGAGTAGGACATGCTGTAGG
ß3-Tub	TGGGCGACTCGGACTTGC	CCACTCTGACCAAAGATGAAATTG

### Flow cytometry

Cells were analysed from cultures at different stages of differentiation (NSC, NPC and mature neurons). Samples for each stage were taken, for NSCs stage when they were at confluence, for NPC stage at the end of the progenitors differentiation (7 days in NPM media) and neuronal stage at DIV40 of the neuronal maturation (cfr. Neuronal differentiation). Briefly, cells for each sample were cultured in a well of 6 MW plate, maintained and differentiated up to the moment of harvesting for immunostainings. Cells were detached by incubating them in accutase and counted (approximately 250.000 cells/well). For surface markers (Cocktail A: CD15-488, CD24-APC, CD44-PE, CD184-PE/Cy, Cocktail B: CD56-450, CD140-PE/Cy and O4-488), the cells were pelleted, resuspended in FACS staining buffer (FSB) (0.5% Fetal Bovine Serum, 2 mM EDTA in PBS), followed by incubation with conjugated antibodies for 30 min on ice. Cells were then washed and pelleted twice with FACS Washing Solution (FWS) discarding the supernatant after every wash. Finally, the cells were resuspended in 200 µL of PBS and analysed on a FACS CANTO. For staining of intracellular markers (Cocktail C: Nestin-PE, GFAP-488, TUJ1-647, Cocktail D: SOX2-488 and Ki67-PE; and independently NeuN or Map2), cells were fixed with the Fixation buffer (FB) (eBioscience) for 40 min in the dark. After fixation, permeabilization buffer (PB) (eBioscience) was added to each tube and cells pelleted for 5 min at 250 g. After discarding the supernatant, cells were washed and pelleted one more time. Cells were then incubated with antibodies dissolved in 100 µL of PB for 60 min in the dark at RT. The cells were then washed once with PB, once with FWS, resuspended in PBS and analysed. For NeuN and Map2 after the wash they were incubated with anti-mouse AF488 for 1 h, washed and analysed. Unstained cell were used as negative control. The FACS CANTO raw data was analysed using FlowJo software.

### Multielectrode array

NPCs were seeded in a 48 MW Multi-Electrode Array (MEA) plate (Axion) at 10.000 cells per well, and differentiated to neurons in NOMc as detailed elsewhere. Activity in each well was recorded at 37 °C for 2 min, every day for 12 weeks (Axion Maestro Middleman). Raw data was processed using the neural metrics tool (AxiS 2.4 software) and exported for further analysis. The general conditions set for the identification of activity were: a) an electrode is active and considered for analysis if #spikes > 1/min, b) spikes were detected when the amplitude was 6.5 SD higher than the noise average, c) a burst (train) consist of a minimum of 5 spikes with a maximum inter-spike interval of 100 ms. Four parameters were taken into account to describe the culture activity and maturation: the mean firing rate of active electrodes per well (Weighted Mean Firing Rate); Number of active electrodes per well; Number of bursts per well; and measure of synchrony between electrodes in a well (Synchrony Index). Three independent experiments with a minimum of four wells each were analyzed.

### Transduction

Neurons grown on microfluidic chambers were transduced with lentiviral particles (LV-mCherry-Mem, LV-GFP, LV-APP-GFP, LV-SYP-GFP, LV-Mito-GFP). Particles were resuspended in NOMc media and after withdrawing the culture media in the somatic compartment, 200 µL were added to the top well. 14–16 h later the transduction media was discarded and replaced with fresh NOMc. After 5 to 7 days of transduction, expression was strong enough for conducting the experiments.

### ELISA quantification

Samples were collected immediately after injury from the flow channel and the axonal compartment of the microfluidic chambers. Non-injured chambers were used as a control. 5–7 chambers were pooled per replicate in order to collect enough volume for the measurements. The proteins were extracted by applying 50 µL of RIPA 1X buffer supplemented with phosphatase and proteinase inhibitors to the axonal compartment well. Cells were scraped with the pipette tip and passed through a 0.8 syringe. Samples were incubated on ice for 30 min before a 20 min centrifugation step at 4 °C, 14.000 RPM.

The protein concentration was assessed via BCA assay (Thermo Scientific™ Pierce™ BCA Protein Assay Kit Catalogue Number: 23227) according to the manufacturer instructions. The concentration between samples was adjusted in order to have the same starting concentration in all the samples.

Samples underwent Enzyme-linked Immunosorbent Assay for quantitative detection of Human Aβ40 and Aβ42 according to the plate manufacturer instructions (Novus Biologicals ELISA kit Human Aβ1-40 cat. NBP2-69,909 and Human Aβ1-42 cat. NBP2-69913).

Briefly, samples and reagents were brought to RT. The standard curve was prepared by making serial dilutions of the standard provided with the kit in duplicate. 100 µL of standards and samples were loaded per well. The plate was incubated for 90 min, at 37 °C in the dark followed by incubation with 100 µL of biotinylated antibody for 1 h at 37 °C in the dark. The plate was then washed 4 times before incubation with HRP-conjugated secondary antibody for 30 min at 37 °C in the dark. 5 washes were performed after the incubation before applying the substrate reagent for 10 min and then the stop solution. The Absorbance was read at 450 nm.

Based on the Absorbance, the concentration in each well was calculated and plotted. Ratios between Aβ42 and Aβ40 were considered to address the impact on Amyloid Beta production in injured axons versus control.

### Proteomics

We performed label free quantitative mass spectrometry in two independent experiments. The first one was a discovery experiment, which consisted in a proteomic analysis, the second one was a confirmatory experiment which consisted in proteomic and phosphoproteomic analysis. Protein extraction protocol was performed in the same way for both experiments. The remaining steps were performed in different facilities as detailed.

#### Protein extraction

Microfluidic chambers of DIV40 were segregated into control and injured group. For each sample, 6–7 chambers were pooled to obtain enough material. Control chambers were subjected to a 10 µL/min flow during 150 s, while injured ones were subjected to 10 µL/min 30 s, 100 µL/min 90 s and 30 µL/min 30 s. Immediately after, chambers were placed on ice, the media was removed completely from all the reservoirs and washed with PBS. 100 µL of lysis buffer (RIPA (10x) Buffer, SDS (10%) 1%, protease inhibitor 1%, phosphatase inhibitor 1%, dH_2_O was added to the top axonal reservoir and flushed trough the axonal compartment to the lower reservoir (repeated 4 times with pipette) to collect all the material from the axonal compartment. The chamber was checked under microscope for efficient collection of axons from the axonal compartment and flow channel. The same procedure was followed for the neuronal compartment. The collection of the first chamber was used to collect 2 more chambers, to avoid dilution of the sample. To disrupt membranes we aspirated the lysate with an insulin syringe for 20 times on ice. Tubes were kept on ice for 30 min and centrifuged at 12,000 g for 20 min at 4 °C. Finally, supernatant was collected, a small volume was used to quantify protein concentration by BCA, and the rest of the sample kept at − 80 °C.

### Discovery experiment

#### Digestion

Samples were reduced with 10 mM dithiothreitol at 60 °C and Alkylated with 50 mM iodoacetamide at RT in the dark. Next, samples were digested with sequencing grade trypsin at 37 °C for 16 h. Digestion was quenched and the peptides collected in formic acid for application to RP-HPLC column. 1 µg of samples was injected to Orbitrap Fusion Lumos and LC–MS/MS data was acquired using designed LC protocol.

#### Mass spectrometry analysis

Trypsin-digested peptides were analyzed by ultra-high pressure liquid chromatography (UPLC) coupled with tandem mass spectroscopy (LC–MS/MS) using nano-spray ionization. The nanospray ionization experiments were performed using an Orbitrap fusion Lumos hybrid mass spectrometer (Thermo) interfaced with nano-scale reversed-phase UPLC (Thermo Dionex UltiMate™ 3000 RSLC nano System). Sample were loaded onto precolumn (volume less than 15 µL, Thermo ACCLAIM PepMap 100 P/N:164564-CMD) at 7 µL/min rate for 5 min followed by an analytical run using a 25 cm, 75 µm ID glass capillary packed with 1.7 µm C18 (130) BEHTM beads (Waters corporation). Peptides were eluted from the C18 column into the mass spectrometer using a linear gradient (5–100%) of ACN (Acetonitrile) at a flow rate of 395 μL/min for 2 h. The buffers used to create the ACN gradient were: Buffer A (98% H_2_O, 2% ACN, 0.1% formic acid) and Buffer B (80% ACN, 0.1% formic acid). Mass spectrometer parameters were as follows: an MS1 survey scan using the orbitrap detector (mass range (m/z): 400–1500 (using quadrupole isolation), 60,000 resolution setting, spray voltage of 2400 V, Ion transfer tube temperature of 290 °C, AGC target of 400,000, and maximum injection time of 50 ms) were followed by data dependent scans (top speed for most intense ions, with charge state set to only include + 2–5 ions, and 20 s exclusion time, while selecting ions with minimal intensities of 50,000 at in which the collision event was carried out in the high energy collision cell (HCD Collision Energy of 30%), and the fragment masses where analyzed in the ion trap mass analyzer (with ion trap scan rate of turbo, first mass m/z was 100, AGC Target 5000 and maximum injection time of 35 ms). Protein identification was carried out using Peaks Studio 8.5 (Bioinformatics solutions Inc.).

#### Data analysis

Data was analyzed using Peaks Studio 8.5. The data search was performed against UNIPROT_Human_9606. The search parameters were set as follows: Search Engine Name: PEAKS; Parent Mass Error Tolerance: 10.0 ppm; Fragment Mass Error Tolerance: 0.4 Da; Precursor Mass Search Type: monoisotopic; Enzyme: Trypsin; Max Missed Cleavages: 3; Digest Mode: Semispecific; Fixed Modifications: Carbamidomethylation: 57.02; Max Variable PTM Per Peptide: 3; Database: Human Uniprot; Taxon: All; Contaminant Database: contaminants; Searched Entry: 21,067; FDR Estimation: Enabled; Merge Options: no merge; Precursor Options: corrected; Charge Options: no correction; Filter Charge: 2–8; Process: true; Associate chimera: yes. The instrument parameters were: Fractions: IP1013_02.raw; Ion Source: ESI(nano-spray); Fragmentation Mode: high energy CID (y and b ions); MS Scan Mode: FT-ICR/Orbitra MS/MS;Scan Mode: Linear Ion Trap.

### Confirmatory experiment

#### Digestion

Protein samples (~ 225 µg) representative of each of the experimental conditions, in four replicates per condition were digested using the FASP protocol [66] with some modifications. Briefly, samples were loaded and buffer exchanged in a 10 kDa MWCO filter unit, reduced with 20 mM tris(2-carboxyethyl)phosphine (TCEP) and alkylated with 100 mM dimethyl acrylamide (DMA). Proteins were digested overnight at 37 °C with Trypsin/Lys-C mix (Mass Spec grade, Promega) at 1:35 enzyme/substrate and shaken at 600 rpm. After tryptic digestion, peptides were collected and extracted with 5% formic acid (FA). Peptide samples were cleaned up using a C18 SPE cartridge (SupelCleanC18, Sigma-Aldrich) and vacuum dried in a Speed Vac. A fraction (~ 25 μg) was kept for direct LC‐MS analysis, and samples stored at– 20 °C.

#### Phosphopeptide enrichment

Phosphorylated peptides were enriched using 5 μL Fe(III)-NTA cartridges and a micro-syringe pump. Dried tryptic peptides ~ 200 μg were dissolved with 30 μL of 0.5% TFA and 120 μL of ACN just before loading the sample to the AssayMAP Fe(III)-NTA cartridges. The cartridges were conditioned with 100 μL 50% ACN, 0.1% TFA at a flow rate of 1200 μL/h and equilibrated with 50 μL loading buffer (80% ACN, 0.1% TFA) at 1200 μL/h. After conditioning, samples were loaded at 350 μL/h onto the cartridge. The cartridge was washed with 50 μL loading buffer follow by a second wash with 50 μL 50% ACN, 0.1% TFA. Phosphorylated peptides were eluted directly into 15 μL of net formic acid in a new 1.5 mL Eppendorf tube with 30 μL of Na_2_HPO_4_, 30 μl of 5% NH_4_OH and 30 μL 5% pyrrolidine, respectively. Pooled elution was cleaned up using a C18 OMIX SPE tip (Agilent, Milford, MA) and dried under low pressure and stored at − 20 °C.

#### Mass spectrometry analysis

Nano-LC MS/MS analysis was performed using an on-line system consisting of a nano-pump UltiMate™ 3000 UHPLC binary HPLC system (Dionex, ThermoFisher) coupled with an Orbitrap Fusion™ Lumos™ Tribrid™ mass spectrometer (ThermoFisher Scientific, Germany) fitted with an EASY-Spray™ nano-electrospray ion source. Whole cell extract peptides and phosphopeptides were resuspended in 1.6% ACN, 0.1% formic acid and injected directly into an EASY-Spray™ PepMap RSLC C18 capillary column (75 µm × 50 cm, 100 Å, 2 μm particle sizes) operated at 50 °C. Peptides were eluted into the MS, at a flow rate of 250 nL/min, using a 136 min gradient as follows: 2% B to 40% B in 120 min followed by ramping to 95% B in 11 min and washing for 5 min. Mobile phase A was 0.1% formic acid in H2O and mobile phase B was 80% acetonitrile and 0.1% formic acid. The mass spectrometer was operated in data-dependent mode, with a single MS scan in the Orbitrap (400–1600 m/z at 60 000 resolution (at 200 m/z) in a profile mode) followed by MS/MS scans in the Orbitrap on the 10 most intense ions at 15,000 resolution. Ions selected for MS/MS scan were fragmented using higher energy collision dissociation (HCD) at normalized collision energy of 27 with an isolation window of 1.4 Th.

#### Data analysis

Mass spectrometry raw data were processed using MaxQuant [67] version 1.6.1.0 with default settings. Dimethyl-Propionamide (C) was set as fixed modification, and acetyl (Protein N-term) and oxidation (M) were allowed as variable modification. The search was performed against the human UniProt database (95,128 sequences, released on October 2020). Protein quantification was based on two or more peptides using the LFQ approach [68]. Proteins were reported at 1% FDR.

MS raw files were loaded to Progenesis LC–MS software (version 4.1, Nonlinear Dynamics, UK) for label free quantification and analysis. Profile data of the MS scans were transformed to peak lists with respective peak m/z values, intensities, abundances and m/z width. MS/MS spectra were treated similarly. For retention time alignment of the samples, the most complex sample was selected as a reference, and the retention times of the other samples were aligned automatically to a maximal overlay of all features. Features with only one charge or more than four charges were excluded from further analyses. Raw abundances of the remaining features were normalized to allow correction for factors resulting from experimental variation.

Rank 1–5 MS/MS spectra were exported as Mascot generic file and used for peptide identification with MASCOT Version 2.4 (Matrix Science Ltd, UK) in the human protein database (Uniprot, 95,128 sequences, released on October 2020). Search parameters were peptide mass tolerance of 10 ppm, and MS/MS tolerance of 0.05 amu allowing 2 missed cleavage. Dimethyl-Propionamide of cysteine was set as a fixed modification, and Oxidation (M), Acetyl (Protein N-term), Phospho (ST) and Phospho (Y) were allowed as variable modification. Peptide assignments with an ion score cut-off of 30 and a significance threshold of *p* < 0.05 were re-imported to Progenesis. After summing up the ion intensities of all the peptides assigned to each protein, a list of proteins abundances was generated. One-way ANOVA was used to calculate the ρ-value based on the abundance values. Results were grouped according to the treatment condition.

### Mass spectrometry data pre-processing

Quality control analyses including correlations, principal components analysis peptide counts and intensities were completed with Perseus (v 1.6.5.0). For the protein abundance analysis one complete experimental set was excluded for the discovery experiment and 2 experimental units (neuronal fraction control and injury) were excluded from the confirmatory experiment, based on outliers in intensities, protein numbers and principal component analysis. Proteins that were positive for “potential contaminant”, “reverse” and “only identified by site” were excluded, as well as a set of proteins that were present in the culture media (i.e., B27/N2 supplements) or extracellular matrix. For each fraction/treatment only proteins that had ≥ 2 peptides identified in at least two replicas were kept (Additional file 10: Table S1). For phosphopeptides only the ones with a score higher than 30 were kept.

PCA, Heatmaps and Volcano were processed in LFQ-Analyst [69] (Fig. 4A, B and Additional file 9: Fig. S3A–C). MaxQuant files were used as input, adjusted p-value cut-off was set to 0.05 and log2 fold change cutoff to 0.585. Perseus-type method was used as missing values imputation method and Benjamini Hochberg for FDR correction.

For phosphoproteomics, peptides with score > 30 were kept. Fold change was calculated with the ratio of the mean normalized abundance for control and injury. P-values and fold change for each phosphopeptide were used for Volcano Plots drawing.

### Enrichment analysis

All enrichment analysis were implemented using g:Profiler [70]. For proteomic analysis two sets of proteins were used: the axonal and the neuronal set. Both of them consisted of the addition of the control and injury treatments set of proteins pre-processed as detailed previously (Additional file 10: Table S1). Over-representation analysis of human tissue specificity was performed using the neuronal protein set as input on the Human Protein Atlas database with a significance threshold of 0.05 and using the g:SCS algorithm for multiple testing correction (Fig. 4C and Additional file 9: Fig. S3D). Gene Ontology Biological Process database was used for functional enrichment analysis for the axonal and neuronal protein set independently with significance threshold of 0.05 and g:SCS for multiple corrections (Fig. 4D). To understand the enrichment of the axonal fraction versus the neuronal fraction, the axonal list of proteins was used as input query and the neuronal one as the background and assessed against the Gene Ontology database. Parameters were set at 0.05 for significance threshold and electronic GO annotations were excluded. The resulting significant terms for Cellular Compartment, Biological Process and Molecular Function were further reduced by exclusion of redundant terms using Revigo [71]. For generating the stringplots the lists of terms for each category were loaded in Cytoscape [72] with all the terms represented as nodes (*p* < 0.05) and the similarity between terms represented as edges (cutoff = 0.3) using EnrichmentMap plugin. Clustering was performed using the AutoAnnotate plugin, with MCL clustering algorithm and labeling for most present words in the nodes (Fig. 4E).

For phosphoproteomics analysis two sets of proteins were considered for each fraction. One set consisted in all the proteins that presented at least one identified phosphopeptide, the other is a subset with proteins that presented at least one phosphopeptide that showed significant changes in abundances between the treatments (*p* < 0.05, fold change > 1.5 or < 0.67). To understand the enrichment of the subset of proteins that significantly change their phosphorylation, this subset was used as input query and the set of all proteins with at least one protein identified was used as the background and assessed against the Gene Ontology Biological Process database (Fig. 5D). This was performed for both the axonal and the neuronal fractions. and assessed against the Gene Ontology database.

Also the subset of proteins that change their phosphorylation in the axonal fraction was used for functional enrichment analysis in the Gene Ontology Molecular Function database and the resulting list reduced by exclusion of redundant terms with Revigo (Fig. 5F).

### Kinase prediction

Specific phosphorylation sites of the axonal protein set with their respective fold changes and significance values were used as the input for the KSEA App [73] with both the PhosphoSitePlus and NetworKIN databases. Three parameters of each kinase were followed: The background-adjusted value of the mean log2(fold change) of all the kinase’s substrates, the number of substrates for each kinase and the statistic significance of the score for each kinase. Kinases with less than two substrates were excluded (Additional file 12: Table S3). Plots were implemented using Coral [74] and Prism.

### Immunofluorescence

Immunofluorescence of the neuronal culture in the microfluidic chambers was performed by first aspirating the media from the wells followed by one wash with PBS and immediate fixing in 4% paraformaldehyde (PFA) in PBS for 45 min at RT. PBS was added to all the wells and the chambers gently peeled off from the coverslip to minimize cell and axonal detachment. Coverslips were sectioned with a diamond pen to isolate the region of interest, and continued with blocking step as next detailed. For immunofluorescence of cells on coverslips, cells were washed with PBS and fixed with 4% PFA in PBS for 30 min at RT, followed by two PBS washes of 10 min each. To avoid non-specific staining, cells were incubated at RT for one hour with blocking solution (10% goat serum, 0.1% Triton X-100 in PBS). Cells were then stained with primary antibodies, dissolved in antibody solution (0.1% Triton X-100 in PBS) and incubated ON at 4 °C. Afterwards, cells were washed twice with PBS and stained with secondary antibodies in antibody solution at RT for 2 h. Finally, cells were stained for 5 min with DAPI and mounted on slides with Mowiol.

Fixed cells were examined with an inverted Zeiss LSM 780 confocal microscope (Zeiss, Germany) using an oil immersion objective (63X/1.4 NA). Images were acquired and processed with Zen Blue and ImageJ softwares, respectively.

### WGA staining

Wheat Germ Agglutinin-Alexa Fluor 633 (Invitrogen) was diluted in PBS (10 µg/mL) and chambers incubated with the solution for 20 min. After one wash with PBS, cells were returned to the growth media for 30 min and imaged.

### Mitochondrial membrane potential

After 5 days of transduction with Mito-GFP, neurons were incubated with tetramethylrhodamine ethyl ester perchlorate (TMRE) (100 nM) diluted in NOM for 30 min. After one wash with PBS they were incubated in aCSF and movies acquired during injury. TMRE intensity was measured during the whole period of the injury and normalized to the intensity of the first 10 frames (F_0_).

### Non-specific membrane permeability

Neurons grown in microfluidic chambers were incubated with a mix of dextranes of different sizes labeled with Cascade Blue (3 KD MW), Alexa Fluor 488 (10 KD MW) and Tetramethylrhodamine (40 KD MW). For this, the dextranes (50 µM in NOM) were loaded in the three lower wells of the microfluidic chambers (80 µL in each well). After injury (10 µL/min × 30 s, 100 µL/min × 90 s, 10 µL/min × 30 s) or control (10 µL/min × 150 s) or positive control treatment (10 µL/min × 150 s, media supplemented with 0.1 mM Triton X-100), chambers were washed three times with aCSF and axonal images taken (40X objective). Intensity of the three different channels inside the axon was measured and a ratio of the intensity inside the axon versus the intensity outside the axon (background) calculated for normalization.

### Calcium transients measurement

NPCs were seeded in multiwell chambers (IBIDI) and differentiated to neurons for 10, 30 and 40 days. They were incubated with the intracellular Ca^2+^ sensor Fluo-4 AM (5 µM in NOMc) for 30 min. After one wash with PBS they were returned to NOMc for 30 min. For imaging, the media was replaced by aCSF. Time lapse movies were recorded at 2 fps for 60 s at 40X/1.3 NA at 480 nm excitation with confocal live module (Zeiss LSM7). In the movies, 10 random projections were selected and intensity analysed using ImageJ. The presence of transients was considered when the maximum intensity levels increased by 10% from the resting levels.

### Axonal ionic levels

Neurons grown in microfluidic chambers were incubated with sodium-binding benzofuran isophthalate (SBFI) (5 µM), FURA-2AM (2 µM) or potassium-binding benzofuran isophthalate (PBFI) (5 µM) in NOM with 0.1% Pluronic for 60 min. Followed by a wash with aCSF, they where returned to NOMc for at least 15 min. Injury was performed in aCSF media. Chambers were mounted in a Zeiss AxioObserver 7 microscope equipped with a HE Fura 2 shift free (E) filter set (Ex BP 340/30, Ex BP 387/15) and imaged with a 40X/1.2 CApo objective. Movies were recorded at 2.5 fps for FURA-2AM and PBFI and at 3.1 fps for SBFI, and axonal intensities of 340 nm and 380 nm measured. After background subtraction for each channel and normalization to the levels of the first 5 frames, the 340/380 nm ratios were calculated.

For control experiments, neurons were grown in IBIDI chambers, incubated with ratiometric probes as described, and incubated in aCSF before imaging. During imaging a solution of KCl (50 mM in aCSF) was perfused.

### Measurements of real-time axonal changes

Movies of axons subjected to the injury were recorded (@1 fps per channel with a 63X/1.4 NA objective). Axons were previously transduced with lentiviral particles containing GFP or mem-mCherry constructs, and in some experiments incubated also with Fluo-4 AM before the injury. To describe the axonal changes, we developed a workflow applied to all the movies. Images were enhanced to maximize the sharpness of the axonal borders. For mCherry-Mem and GFP, where intensity measurements were not performed, CLAHE filter followed by Top-hat transformations were applied, while for the Fluo-4 AM only linear filters were applied. After the pre-processing step, axonal longitudinal axis was divided into approximately 30 columns with a fixed width (from 1.5 to 3 µm depending on the total axonal length), defining 30 segments. Next, the enhanced image was binarized using Otsu’s threshold that minimizes the weighted within-class variance. For each of the segments a ROI consisting of the two axon shaft borders (i.e. the “upper” and the “lower”) was detected. Thus, all the segments of the axon could be tracked independently during the movie. In this way, for each segment, the distance between one border and the other (thickness) was computed throughout the movie. An axonal swelling is a histopathological hallmark where axons get swollen (enlarged) in a particular region. Thus for detecting AS, we searched for segmental increases in thickness. In a given frame the median thickness of all the segments was calculated resulting in the median thickness of the axon at a given time and set as 100%. From this, the segments that present a thickness of 150% or higher were identified and considered as AS. By iterating this operation in every frame of the movie the development of AS can be tracked in space and time. In the analysis, where number of AS per axons was studied, any AS that was present in the pre-injury stage was subtracted to the whole movie for normalization purposes.

For the Ca^2+^ levels analysis in conjunction with the membrane changes, Fluo-4 AM intensity levels were assessed in the same ROIs delimited by the membrane marker (mem-mCherry). Thus, for every segment of the axon we attributed an intensity. For analysis of variation of Ca^2+^ levels through time, intensity levels were normalized to the pre-injury stage levels (mean of first 30 frames). To assess the presence of high Ca^2+^ levels in the AS we analysed the matrix (N x T) of the axons which consisted of T frames (length of movie in s) and N segments (number of segments dividing the axon). For each coordinate (n,t) we assigned presence/absence of AS, and presence/absence of high Ca^2+^. AS was present at (n,t) if the thickness value in (n,t) was ≥ 1.5 × the median thickness value of all n at time t. A high Ca^2+^ level was assigned at (n,t) if the Ca^2+^ level value in (n,t) was ≥ 1.25 × the mean Ca^2+^ value of the first 30 t in that n. This Ca^2+^ normalization was made for establishing the Ca^2+^ resting levels at the initial period of the acquisition (F0). Swelling detection failed in some cases (n,t) due to loss of focus or errors in the detection algorithm. This led to discontinuity in the tracking of some AS (eg. in a given segment, from 50 to 60 s a swelling was detected but s 61 and 62 was not, followed by 63–80 with swelling). To not overrepresent the AS numbers, we applied a formula to connect these AS. Thus, if in a given segment the AS had a duration of *l* frames, it merged the following AS only if this was not further than 0.3 × *l*. After this processing, we performed the analysis of high Ca^2+^ levels throughout the duration of the AS, with 3 possible categories: high Ca^2+^ levels were present during all the duration of the swelling, high Ca^2+^ was not present in any moment of the duration of the swelling, high Ca^2+^ levels were present during some moments of the AS duration. To assess if high Ca^2+^ precedes the AS formation, we analyzed the Ca^2+^ levels of the two frames preceding (2 s) the AS.

### Pharmacological blockage of calcium stores

Dose–response experiments were performed to set the drug concentration to use in injury. Neuronal cultures DIV 40 grown in IBIDI chambers were incubated with Fluo-4AM (5 µM in NOMc) for 30 min. Based on previous literature we chose a range of concentrations for every drug and incubated the cultures for 30–60 min in NOMc. After incubation, drugs were maintained in all solutions. Cultures were kept in aCSF and a solution of KCl (50 mM in aCSF) applied. Time lapse movies were recorded at 2 fps for 300 s at 40X/1.3 NA at 480 nm excitation with confocal live module (Zeiss LSM7). First 100 s were used for normalization purposes and were followed by the application of KCl. The mean Fluo-4AM intensity of 15 projections was measured during the treatment, followed by the calculation of the area under the curve.

Axonal injury with pharmacological blockage of Ca^2+^ stores was performed in microfluidic chambers following the same procedure as detailed before, with minor changes. Briefly, DIV 40 neurons in microfluidic chambers were transduced with mem-mCherry. After 5 days, neurons were incubated with Fluo-4AM for 30 min. Neurons were then incubated with Nifedipine (1 µM), Gadolinium (100 µM), Ryanodine (100 µM), Xestospongin C (10 µM) or CGP317157 (10 µM) in NOMc for 30–60 min. Following incubation compounds were kept in all medias. Incubation was followed by a wash in aCSF and kept in aCSF for imaging. Movies of axons subjected to the injury protocol were recorded (@1 fps per channel with a 63X/1.4 NA objective). Number of AS and Ca^2+^ intensities during time were calculated as detailed previously. During and post-injury calculations of mean number of AS and mean Ca levels were done by computing the last 30 s of the injury period and the last 30 s of the post-injury period.

### Axonal transport

The movement dynamics of APP-GFP, Synaptophysin-GFP and Mito-GFP particles was analyzed using the same protocol. Movies of both control axons and those subjected to injury were acquired at 1fps for Synaptophysin and Mito, and at @2fps for APP, in a confocal microscope equipped with a live module (Zeiss Confocal LSM780, Zeiss Live LSM7) with an immersion oil objective 63x/1.4 NA Plan Apochromat. Time-lapse movies were processed with ImageJ, prior to their analysis in Imaris. Particles were tracked with semi-automated spot tracking algorithm and visualized during the whole period of the movie. The identified movement of each particle during the movie were represented as spots and tracks. First, we choosed the algorithm that would define the movement of our cargoes of interest in the best way. Considering that our cargoes have a complete or almost continuous movement, we applied an Autoregressive Motion algorithm. This semi-automated algorithm requires the input of the following parameters: XY estimated diameter, max distance, and max gap size. Diameter was based on an average empirical value for each specific cargo analysed. For both max distance and max gap size we considered both spatial and temporal resolution before applying any kind of value. Movement along all the axes of the axon was analyzed, but just that along the *x* axis was taken into account. Among all the detailed statistics obtained from the movies analysis, the most significant one is the spatial displacement of a particle in each frame, which was exported for further computation of different axonal transport parameters. With this value we decided to analyze stationary vs moving tracks, average velocities, and time proportions of anterograde/retrograde/stationary during 3 periods: (1) pre-injury, (2) injury, (3) post-injury. For transport dynamics analysis we first quantified the tracks in stationary or moving status. The stationary tracks were those that didn’t show any movement in the whole period (pre-injury, injury and post-injury) of the movie, presenting an average velocity < 0.010 µm/s. These tracks were excluded from the analysis. For the moving tracks we first set a threshold based on track duration (td = total time during which a particle moves) choosing only those lasting > 10 s. Among the remaining tracks we took for each stage (pre-injury, injury, post-injury) the mean displacement over time. Thus, the movement of the particle for each independent stage was considered anterograde or retrograde if the average velocity was > 0.01 µm/s or < − 0.01 µm/s, respectively, otherwise they were considered stationary.

### Structured illumination microscopy

Samples were fixed and immunofluorescence was performed as stated elsewhere. Secondary antibodies used for cytoskeletal proteins were conjugated with Alexa-488 and secondary antibody used for mCherry was conjugated with Alexa-555. Samples were examined in a Zeiss Axioimager Z.1 platform equipped with the Elyra PS.1 super-resolution module for structured illumination (SIM). The high-resolution images were acquired in super-resolution mode using Zeiss Pln Apo 63x/1.4 Oil objective (tot. mag. 1008x) with appropriate immersion oil (Immersol 518F). The SR-SIM setup involved 5 rotations and 5 phases. The software recommended grating was used for each fluorescence channel. For each image, up to 30 Z-stacks (101 nm) were acquired. Image acquisitions and SIM processing were performed in Zeiss Zen 11 software.

### Actin and βII-Spectrin periodicity analysis

Z-stacks of actin or βII-Spectrin stained axons and mem-mCherry acquired by SIM were analysed in IMARIS v9. Specific ROIs (shaft, swellings) were cropped and two independent procedures were applied to each channel. The mem-mCherry channel was used to define by auto-thresholding the surface of the AS or the shaft. The channel of the actin or the βII-Spectrin was used to define spots with auto-threshold. All the spots that were outside the volume set with mem-mCherry channel were removed, by using the “spots close to surface” command. The distance between the spots and their closest neighbour was measured with the “spot to spot closest distance” command. Measurements and quantitative data was exported and processed in Excel or Prism.

Validation of the method was conducted by comparing the IMARIS closest neighbour results with intensity plots that result from the trace of a longitudinal line to the mayor axis of the axon shaft [75]. For the latter, the distances between peaks of the intensity profile were plotted as a frequency distribution. This frequency distribution was similar to the frequency distribution obtained by the closest neighbour method (Additional file 13: Fig. S4B).

### Scanning electron microscopy imaging

Following injury, chambers were washed with PBS and all the wells filled with fixation solution (Glutaraldehyde 6%, Sodium Cacodylate Buffer 0.2 M pH 7.35, 1:1) for 1 h at RT. The chambers were washed with Cacodylate Buffer, and dehydrated in a series of increasing concentrations of ethanol (2 × 10 min 50°, 2 × 20 min 80°, 2 × 20 min 100° EtOH). Chambers were separated from the coverslip by peeling off and coverslips cut with a diamond pen to isolate the region of interest. Samples were left to dry and coated with an automatic sputter coater (JEOL JFC-1300) for 15 s at 40 mA for enhancing the contrast. Imaging was performed in a JEOL JCM-6000 scanning electron microscope at high vacuum mode, secondary electron image and accelerating voltage of 10 kV.

AS lengths and widths were measured in SEM images of control and injured axons with ImageJ. Inter-swelling distances were measured between the end of AS to the beginning of the adjacent one. Percentage of number of AS per axons were fitted by using a Gaussian model with least squares regression on Prism 9. Relative frequencies of inter-swelling distances were modelled as a Gaussian mixture with parameters determined by the maximum-likelihood estimate (MLE) via the expectation–maximization (EM) algorithm as implemented in the R-package mclust.

### Transmission electron microscopy

Following injury, chambers were washed with PBS and fixed with 2.5% glutaraldehyde and 2% PFA in 0.1 M phosphate buffer (pH 7.4) for 2 h at RT. After fixation, chambers were gently peeled off from the coverslips. Contrast enhancement with 1% OsO_4_ in phosphate buffer for 30 min and 1% uranyl acetate in 50% ethanol/water for 30 min was performed. Samples were then dehydrated in a graded ethanol series and embedded in Durcupan. Ultrathin sections (70 nm) were sliced using a microtome (EM UC7 Ultramicrotome, Leica Microsystems) and mounted on formvar coated copper slot grids. Images were acquired with a transmission electron microscope (Tecnai 10, FEI; operated at 80 kV, equipped with OSIS Megaview III camera).

### Antibodies and Reagents

Antibodies, vectors, reagents, kits and software can be found in Additional file 20.

### Quantification and statistical analysis

Graphpad Prism 9.3.0 was used for all statistical analyses. Statistical tests are as described in text and figure legends.

## Supplementary Information

**Additional file 1: Fig. S1.** Characterization of injury system, Related to Fig. 1.

**Additional file 2: Video S1.** Fluorescent beads in the flow channel of the microfluidic chamber during a pump flow rate of 100 µL/min. Video recorded at 800 fps using brightfield and epifluorescence illumination.

**Additional file 3: Video S2.** Urethane pillar placed in the flow channel of the microfluidic chamber during a pump flow rate of 400 µL/min. Video recorded at 5 fps using brightfield illumination.

**Additional file 4: Video S3.** Ca^2+^ transients of 40 DIV neuronal cultures incubated with Fluo-4AM. Video recorded using 489 nm laser at 1 fps.

**Additional file 5: Video S4.** Axon from mem-mCherry transduced neuronal cultures subjected to injury. Segmentation and identification of normal shaft (blue) and swelling (red) regions. Video recorded using 561 nm laser at 1 fps. Inverted grayscale.

**Additional file 6: Video S5.** Axon from GFP transduced neuronal cultures subjected to injury. Segmentation and identification of normal shaft (blue) and swelling (red) regions. Video recorded using 489 nm laser at 1 fps. Inverted grayscale.

**Additional file 7: Fig. S2.** Membrane permeability and Ca^2+^ blockers dose response assessment, Related to Fig. 3.

**Additional file 8: Video S6.** Axon subjected to injury from mem-mCherry transduced and Fluo-4AM incubated neuronal cultures. For both channels, segmentation and identification of normal shaft (blue) and swelling (red) regions. Video recorded using 489 nm and 561 lasers at 1 fps per channel. Inverted grayscale.

**Additional file 9: Fig. S3.** Axonal proteomic profiling before and immediately after injury, Related to Fig. 4.

**Additional file 10: Table S1.** Proteomics original and processed data for both discovery and confirmatory experiments.

**Additional file 11: Table S2.** Phosphoproteomics original data and detailed phosphorylation information.

**Additional file 12: Table S3.** Kinase analysis results.

**Additional file 13: Fig. S4.** Structural reorganization of the axonal cytoskeleton following injury, Related to Fig. 6.

**Additional file 14: Video S7.** Axon from CAMSAP2-GFP transfected neuronal cultures subjected to injury. Video recorded using 489 nm laser at 1 fps. Inverted grayscale. Scale bar: 10 µm.

**Additional file 15: Video S8.** Axon from APP-GFP transduced neuronal culture subjected to injury. Tracking of APP-GFP vesicles was performed during the whole video using IMARIS. Video recorded using 489 nm laser at 2 fps.

**Additional file 16: Video S9.** APP-GFP vesicles movement dynamics through an axonal swelling. Video recorded using 489 nm laser at 2 fps.

**Additional file 17: Video S10.** Axon from Synaptophysin-GFP transduced neuronal culture subjected to injury. Tracking of Synaptophysin-GFP vesicles was performed during the whole video using IMARIS. Video recorded using 489 nm laser at 1 fps.

**Additional file 18: Video S11.** Axon from Mito-GFP transduced neuronal culture subjected to injury. Tracking of Mito-GFP vesicles was performed during the whole video using IMARIS. Video recorded using 489 nm laser at 2 fps.

**Additional file 19: Video S12.** Axon subjected to injury from Mito-GFP (green) transduced neuronal cultures followed by TMRE (red) incubation. Video recorded using 489 nm and 561 lasers at 2 fps per channel.

**Additional file 20**. Key Resources Table: Table including antibodies, resources, kits, cells, and software with catalog numbers.

## Data Availability

All data are available in the supplementary files.

## References

[1] Faul M, Coronado V (2015). Epidemiology of traumatic brain injury. Handb Clin Neurol.

[2] Rand CW, Courville CB (1946). Histologic changes in the brain in cases of fatal injury to the head; alterations in nerve cells. Arch Neurol Psychiatry.

[3] Ziogas NK, Koliatsos VE (2018). Primary traumatic axonopathy in mice subjected to impact acceleration: a reappraisal of pathology and mechanisms with high-resolution anatomical methods. J Neurosci.

[4] Reeves TM, Phillips LL, Povlishock JT (2005). Myelinated and unmyelinated axons of the corpus callosum differ in vulnerability and functional recovery following traumatic brain injury. Exp Neurol.

[5] Geula C, Nagykery N, Nicholas A, Wu CK (2008). Cholinergic neuronal and axonal abnormalities are present early in aging and in Alzheimer disease. J Neuropathol Exp Neurol.

[6] Yagishita S, Kimura S (1975). Infantile neuroaxonal dystrophy (Seitelberger’s disease). A light and ultrastructural study. Acta Neuropathol.

[7] Stokin GB, Goldstein LSB (2006). Axonal transport and Alzheimer’s disease. Annu Rev Biochem.

[8] Henninger N, Bouley J, Sikoglu EM, An J, Moore CM, King JA (2016). Attenuated traumatic axonal injury and improved functional outcome after traumatic brain injury in mice lacking Sarm1. Brain.

[9] Hånell A, Greer JE, McGinn MJ, Povlishock JT (2015). Traumatic brain injury-induced axonal phenotypes react differently to treatment. Acta Neuropathol.

[10] Edwards G, Zhao J, Dash PK, Soto C, Moreno-Gonzalez I (2020). Traumatic brain injury induces tau aggregation and spreading. J Neurotrauma.

[11] Saatman KE, Abai B, Grosvenor A, Vorwerk CK, Smith DH, Meaney DF (2003). Traumatic axonal injury results in biphasic calpain activation and retrograde transport impairment in mice. J Cereb Blood Flow Metab.

[12] Jassam YN, Izzy S, Whalen M, McGavern DB, El Khoury J (2017). Neuroimmunology of traumatic brain injury: time for a paradigm shift. Neuron.

[13] Kant A, Johnson VE, Arena JD, Dollé JP, Smith DH, Shenoy VB (2021). Modeling links softening of myelin and spectrin scaffolds of axons after a concussion to increased vulnerability to repeated injuries. Proc Natl Acad Sci U S A.

[14] Ahluwalia M, Kumar M, Ahluwalia P, Rahimi S, Vender JR, Raju RP (2021). Rescuing mitochondria in traumatic brain injury and intracerebral hemorrhages: a potential therapeutic approach. Neurochem Int.

[15] Datar A, Ameeramja J, Bhat A, Srivastava R, Mishra A, Bernal R (2019). The roles of microtubules and membrane tension in axonal beading, retraction, and atrophy. Biophys J.

[16] Tang-Schomer MD, Patel AR, Baas PW, Smith DH (2010). Mechanical breaking of microtubules in axons during dynamic stretch injury underlies delayed elasticity, microtubule disassembly, and axon degeneration. FASEB J.

[17] Chen XH, Meaney DF, Xu BN, Nonaka M, McIntosh TK, Wolf JA (1999). Evolution of neurofilament subtype accumulation in axons following diffuse brain injury in the pig. J Neuropathol Exp Neurol.

[18] Cross DJ, Meabon JS, Cline MM, Richards TL, Stump AJ, Cross CG (2019). Paclitaxel reduces brain injury from repeated head trauma in mice. J Alzheimers Dis.

[19] Tang-Schomer MD, Johnson VE, Baas PW, Stewart W, Smith DH (2012). Partial interruption of axonal transport due to microtubule breakage accounts for the formation of periodic varicosities after traumatic axonal injury. Exp Neurol.

[20] DiLeonardi AM, Huh JW, Raghupathi R (2009). Impaired axonal transport and neurofilament compaction occur in separate populations of injured axons following diffuse brain injury in the immature rat. Brain Res.

[21] Beirowski B, Nógrádi A, Babetto E, Garcia-Alias G, Coleman MP (2010). Mechanisms of axonal spheroid formation in central nervous system Wallerian degeneration. J Neuropathol Exp Neurol.

[22] Borgens RB, Jaffe LF, Cohen MJ (1980). Large and persistent electrical currents enter the transected lamprey spinal cord. Proc Natl Acad Sci U S A.

[23] Barsukova AG, Forte M, Bourdette D (2012). Focal increases of axoplasmic Ca^2+^, aggregation of sodium-calcium exchanger, N-type Ca^2+^ channel, and actin define the sites of spheroids in axons undergoing oxidative stress. J Neurosci.

[24] Staal JA, Dickson TC, Gasperini R, Liu Y, Foa L, Vickers JC (2010). Initial calcium release from intracellular stores followed by calcium dysregulation is linked to secondary axotomy following transient axonal stretch injury. J Neurochem.

[25] Stirling DP, Cummins K, Wayne Chen SR, Stys P (2014). Axoplasmic reticulum Ca^2+^ release causes secondary degeneration of spinal axons. Ann Neurol.

[26] Gu Y, Jukkola P, Wang Q, Esparza T, Zhao Y, Brody D (2017). Polarity of varicosity initiation in central neuron mechanosensation. J Cell Biol.

[27] Yuen TJ, Browne KD, Iwata A, Smith DH (2009). Sodium channelopathy induced by mild axonal trauma worsens outcome after a repeat injury. J Neurosci Res.

[28] Wolf JA, Stys PK, Lusardi T, Meaney D, Smith DH (2001). Traumatic axonal injury induces calcium influx modulated by tetrodotoxin-sensitive sodium channels. J Neurosci.

[29] Ribas VT, Koch JC, Michel U, Bähr M, Lingor P (2017). Attenuation of axonal degeneration by calcium channel inhibitors improves retinal ganglion cell survival and regeneration after optic nerve crush. Mol Neurobiol.

[30] Hemphill MA, Dabiri BE, Gabriele S, Kerscher L, Franck C, Goss JA (2011). A possible role for integrin signaling in diffuse axonal injury. PLoS ONE.

[31] Dubreuil CI, Marklund N, Deschamps K, McIntosh TK, McKerracher L (2006). Activation of Rho after traumatic brain injury and seizure in rats. Exp Neurol.

[32] Chung RS, Staal JA, McCormack GH, Dickson TC, Cozens MA, Chuckowree JA (2005). Mild axonal stretch injury in vitro induces a progressive series of neurofilament alterations ultimately leading to delayed axotomy. J Neurotrauma.

[33] Garland P, Broom LJ, Quraishe S, Dalton PD, Skipp P, Newman TA (2012). Soluble axoplasm enriched from injured CNS axons reveals the early modulation of the actin cytoskeleton. PLoS ONE.

[34] Nijssen J, Aguila J, Hoogstraaten R, Kee N, Hedlund E (2018). Axon-Seq decodes the motor axon transcriptome and its modulation in response to ALS. Stem Cell Rep.

[35] Taylor AM, Dieterich DC, Ito HT, Kim SA, Schuman EM (2010). Microfluidic local perfusion chambers for the visualization and manipulation of synapses. Neuron.

[36] Vugmeyster L, McKnight CJ (2009). Phosphorylation-induced changes in backbone dynamics of the dematin headpiece C-terminal domain. J Biomol NMR.

[37] Juanes-Garcia A, Chapman JR, Aguilar-Cuenca R, Delgado-Arevalo C, Hodges J, Whitmore LA (2015). A regulatory motif in nonmuscle myosin II-B regulates its role in migratory front-back polarity. J Cell Biol.

[38] Sun C, Zheng J, Cheng S, Feng D, He J (2013). EBP50 phosphorylation by Cdc2/Cyclin B kinase affects actin cytoskeleton reorganization and regulates functions of human breast cancer cell line MDA-MB-231. Mol Cells.

[39] Devaux S, Poulain FE, Devignot V, Lachkar S, Irinopoulou T, Sobel A (2012). Specific serine-proline phosphorylation and glycogen synthase kinase 3β-directed subcellular targeting of stathmin 3/Sclip in neurons. J Biol Chem.

[40] Xu K, Zhong G, Zhuang X (2013). Actin, spectrin, and associated proteins form a periodic cytoskeletal structure in axons. Science.

[41] Chen XH, Siman R, Iwata A, Meaney DF, Trojanowski JQ, Smith DH (2004). Long-term accumulation of amyloid-beta, beta-secretase, presenilin-1, and caspase-3 in damaged axons following brain trauma. Am J Pathol.

[42] Serbest G, Horwitz J, Barbee K (2005). The effect of poloxamer-188 on neuronal cell recovery from mechanical injury. J Neurotrauma.

[43] Kilinc D, Gallo G, Barbee KA (2008). Mechanically-induced membrane poration causes axonal beading and localized cytoskeletal damage. Exp Neurol.

[44] Greer JE, Hånell A, McGinn MJ, Povlishock JT (2013). Mild traumatic brain injury in the mouse induces axotomy primarily within the axon initial segment. Acta Neuropathol.

[45] Pullarkat PA, Dommersnes P, Fernández P, Joanny JF, Ott A (2006). Osmotically driven shape transformations in axons. Phys Rev Lett.

[46] Costa AR, Sousa SC, Pinto-Costa R, Mateus JC, Lopes CDF, Costa AC (2020). The membrane periodic skeleton is an actomyosin network that regulates axonal diameter and conduction. eLife.

[47] Fan A, Tofangchi A, Kandel M, Popescu G, Saif T (2017). Coupled circumferential and axial tension driven by actin and myosin influences in vivo axon diameter. Sci Rep.

[48] Charras GT, Hu CK, Coughlin M, Mitchison TJ (2006). Reassembly of contractile actin cortex in cell blebs. J Cell Biol.

[49] Hannemann S, Madrid R, Stastna J, Kitzing T, Gasteier J, Schönichen A (2008). The Diaphanous-related Formin FHOD1 associates with ROCK1 and promotes Src-dependent plasma membrane blebbing. J Biol Chem.

[50] Larsson C (2006). Protein kinase C and the regulation of the actin cytoskeleton. Cell Signal.

[51] Wayman GA, Kaech S, Grant WF, Davare M, Impey S, Tokumitsu H (2004). Regulation of axonal extension and growth cone motility by calmodulin-dependent protein kinase I. J Neurosci.

[52] Tararuk T, Ostman N, Li W, Björkblom B, Padzik A, Zdrojewska J (2006). JNK1 phosphorylation of SCG10 determines microtubule dynamics and axodendritic length. J Cell Biol.

[53] Reinhardt L, Kordes S, Reinhardt P, Glatza M, Baumann M, Drexler HCA (2019). Dual Inhibition of GSK3β and CDK5 protects the cytoskeleton of neurons from neuroinflammatory-mediated degeneration in vitro and in vivo. Stem Cell Rep.

[54] Amano M, Nakayama M, Kaibuchi K (2010). Rho-kinase/ROCK: a key regulator of the cytoskeleton and cell polarity. Cytoskeleton (Hoboken).

[55] Leite SC, Sampaio P, Sousa VF, Nogueira-Rodrigues J, Pinto-Costa R, Peters LL (2016). The actin-binding protein α-adducin is required for maintaining axon diameter. Cell Rep.

[56] Chaves RS, Tran M, Holder AR, Balcer AM, Dickey AM, Roberts EA (2021). Amyloidogenic processing of amyloid precursor protein drives stretch-induced disruption of axonal transport in hiPSC-derived neurons. J Neurosci.

[57] Lang-Ouellette D, Gruver KM, Smith-Dijak A, Blot FGC, Stewart CA, de Vanssay de Blavous P (2021). Purkinje cell axonal swellings enhance action potential fidelity and cerebellar function. Nat Commun.

[58] Babij R, Lee M, Cortés E, Vonsattel J-PG, Faust PL, Louis ED (2013). Purkinje cell axonal anatomy: quantifying morphometric changes in essential tremor versus control brains. Brain.

[59] Marion CM, Radomski KL, Cramer NP, Galdzicki Z, Armstrong RC (2018). Experimental traumatic brain injury identifies distinct early and late phase axonal conduction deficits of white matter pathophysiology, and reveals intervening recovery. J Neurosci.

[60] von Reyn CR, Mott RE, Siman R, Smith DH, Meaney DF (2012). Mechanisms of calpain mediated proteolysis of voltage gated sodium channel α-subunits following in vitro dynamic stretch injury. J Neurochem.

[61] Johnson VE, Stewart W, Smith DH (2013). Axonal pathology in traumatic brain injury. Exp Neurol.

[62] Rudrabhatla P, Grant P, Jaffe H, Strong MJ, Pant HC (2010). Quantitative phosphoproteomic analysis of neuronal intermediate filament proteins (NF-M/H) in Alzheimer’s disease by iTRAQ. FASEB J.

[63] Rudrabhatla P, Jaffe H, Pant HC (2011). Direct evidence of phosphorylated neuronal intermediate filament proteins in neurofibrillary tangles (NFTs): phosphoproteomics of Alzheimer’s NFTs. FASEB J.

[64] Plassman BL, Havlik RJ, Steffens DC, Helms MJ, Newman TN, Drosdick D (2000). Documented head injury in early adulthood and risk of Alzheimer’s disease and other dementias. Neurology.

[65] Schoen I, Hu W, Klotzsch E, Vogel V (2010). Probing cellular traction forces by micropillar arrays: contribution of substrate warping to pillar deflection. Nano Lett.

[66] Wiśniewski JR, Zougman A, Nagaraj N, Mann M (2009). Universal sample preparation method for proteome analysis. Nat Methods.

[67] Cox J, Mann M (2008). MaxQuant enables high peptide identification rates, individualized p.p.b.-range mass accuracies and proteome-wide protein quantification. Nat Biotechnol.

[68] Cox J, Hein MY, Luber CA, Paron I, Nagaraj N, Mann M (2014). Accurate proteome-wide label-free quantification by delayed normalization and maximal peptide ratio extraction, termed MaxLFQ. Mol Cell Proteom.

[69] Shah AD, Goode RJA, Huang C, Powell DR, Schittenhelm RB (2020). LFQ-analyst: an easy-to-use interactive web platform to analyze and visualize label-free proteomics data preprocessed with MaxQuant. J Proteome Res.

[70] Raudvere U, Kolberg L, Kuzmin I, Arak T, Adler P, Peterson H (2019). g:Profiler: a web server for functional enrichment analysis and conversions of gene lists (2019 update). Nucleic Acids Res.

[71] Supek F, Bošnjak M, Škunca N, Šmuc T (2011). REVIGO summarizes and visualizes long lists of gene ontology terms. PLoS ONE.

[72] Shannon P, Markiel A, Ozier O, Baliga NS, Wang JT, Ramage D (2003). Cytoscape: a software environment for integrated models of biomolecular interaction networks. Genome Res.

[73] Casado P, Rodriguez-Prados JC, Cosulich SC, Guichard S, Vanhaesebroeck B, Joel S (2013). Kinase-substrate enrichment analysis provides insights into the heterogeneity of signaling pathway activation in leukemia cells. Sci Signal.

[74] Metz KS, Deoudes EM, Berginski ME, Jimenez-Ruiz I, Aksoy BA, Hammerbacher J (2018). Coral: clear and customizable visualization of human kinome data. Cell Syst.

[75] D’Este E, Kamin D, Velte C, Göttfert F, Simons M, Hell SW (2016). Subcortical cytoskeleton periodicity throughout the nervous system. Sci Rep.

