# Supplementary material for: Unraveling axonal mechanisms of traumatic brain injury
Source: Acta Neuropathol Commun. 2022 Sep 21;10:140. doi: 10.1186/s40478-022-01414-8 (PMC9494812; doi:10.1186/s40478-022-01414-8)
Supplement: Supplementary file 1 — Additional file 1: Fig. S1. Characterization of injury system, Related to Fig. 1. [file 40478_2022_1414_MOESM1_ESM.pdf]

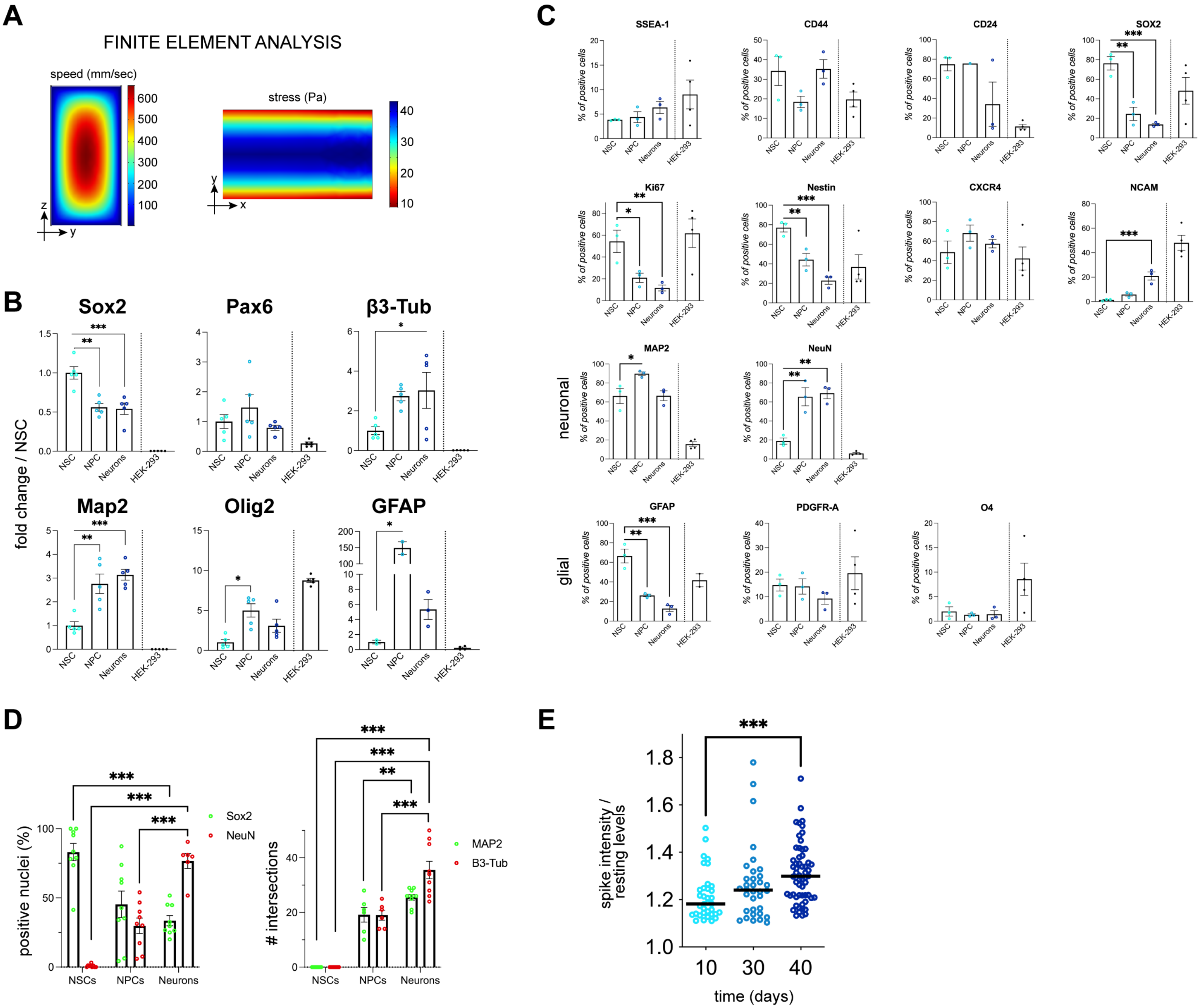

Figure S1. Characterization of injury system, Related to Figure 1.
(A) Finite element analysis method applied to a simplified version of the microfluidic chamber used to predict the speed and stress on the injury channel.
(B) RT-PCR analysis of mRNA expression levels of markers of early stages, neuronal and glial lineage through differentiation of NSCs to mature neurons. HEK-293 cells were used as a control cell line (n=5).
(C) Quantification of positive cells by flow cytometry for different markers of neuronal lineage through differentiation of NSCs to mature neurons. HEK-293 cells were used as a control cell line (n=3).
(D) Quantification of immunofluorescence stainings against Sox2 and NeuN or Map2 and ß3Tub in NSC, NPC and neuronal stages (n=2, >6 images/n). Number of intersections found from ß3-Tub or Map2 positive projections was evaluated
(E) Spike intensityies of Ca2+ transients (n>34 axons).
Data are mean±SEM (B,C and D) or median (E) (*p<0.05, **p<0.01, ***p<0.001). Statistical comparisons were performed using repeated measures one-way ANOVA followed by Dunnett's multiple comparisons test (B and C), or a one way ANOVA followed by Dunnett's multiple comparisons test (D), or Kruskal Wallis test followed by a Dunn's multiple comparison test (E).
