# Supplementary material for: Unraveling axonal mechanisms of traumatic brain injury
Source: Acta Neuropathol Commun. 2022 Sep 21;10:140. doi: 10.1186/s40478-022-01414-8 (PMC9494812; doi:10.1186/s40478-022-01414-8)
Supplement: Supplementary file 7 — Additional file 7: Fig. S2. Membrane permeability and Ca2+ blockers dose response assessment, Related to Fig. 3. [file 40478_2022_1414_MOESM7_ESM.pdf]

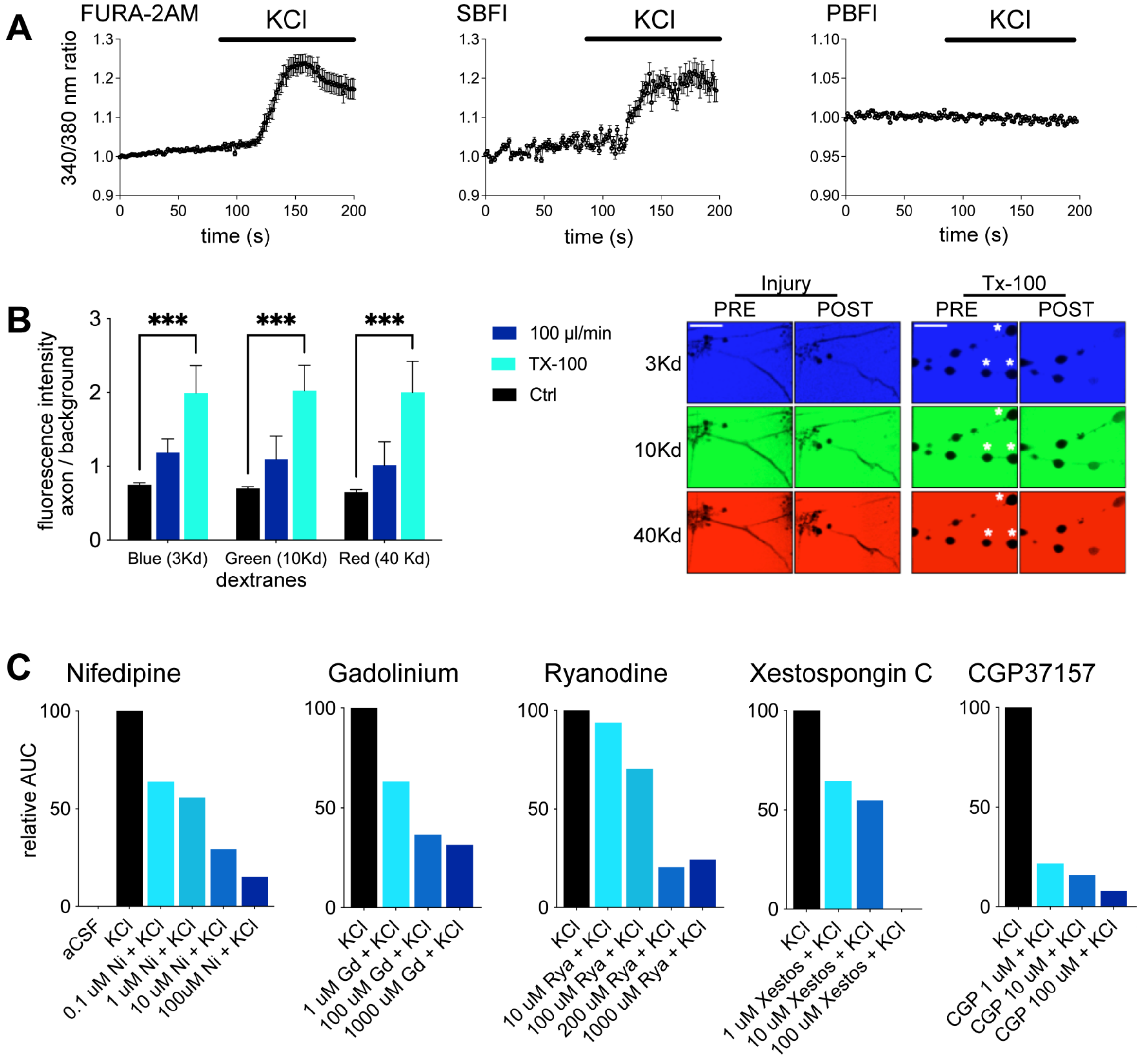

Figure S2. Membrane permeability and Ca2+ blockers dose response assessment, Related to Figure 3.

(A) Neuronal cultures were perfused with KCl-aCSF to assess the response of the ratiometric probes. PBFI lack of response is due to equilibration of inner and outer K+ concentrations after KCl application (n=15 axons).

(B) Permeability of dextranes of different sizes in the axon after injury. Bar graph shows the ratio between the mean fluorescence intensity inside the axon over the mean intensity in adjacent media for each dextrane. Representative micrographs showing the dextranes intensity levels inside and outside the axons, before or after the injury (treatment). Note how parts of the axons (asterisks) get filled with dextranes present in the media(n=3, 7 axons/n, scale bar: 20 µm).

(C) Dose response curves of Ca2+ blocker compounds in neuronal cultures. Fluo-4AM intensity was measured and area under the curve calculated after application of 50 mM KCl (n=15 axons).

Data represented as mean +SEM (***p<0.001). A two way ANOVA was performed followed by a Dunnett's multiple comparisons test versus the control treatment (B).
