# Supplementary material for: Unraveling axonal mechanisms of traumatic brain injury
Source: Acta Neuropathol Commun. 2022 Sep 21;10:140. doi: 10.1186/s40478-022-01414-8 (PMC9494812; doi:10.1186/s40478-022-01414-8)
Supplement: Supplementary file 9 — Additional file 9: Fig. S3. Axonal proteomic profiling before and immediately after injury, Related to Fig. 4. [file 40478_2022_1414_MOESM9_ESM.pdf]

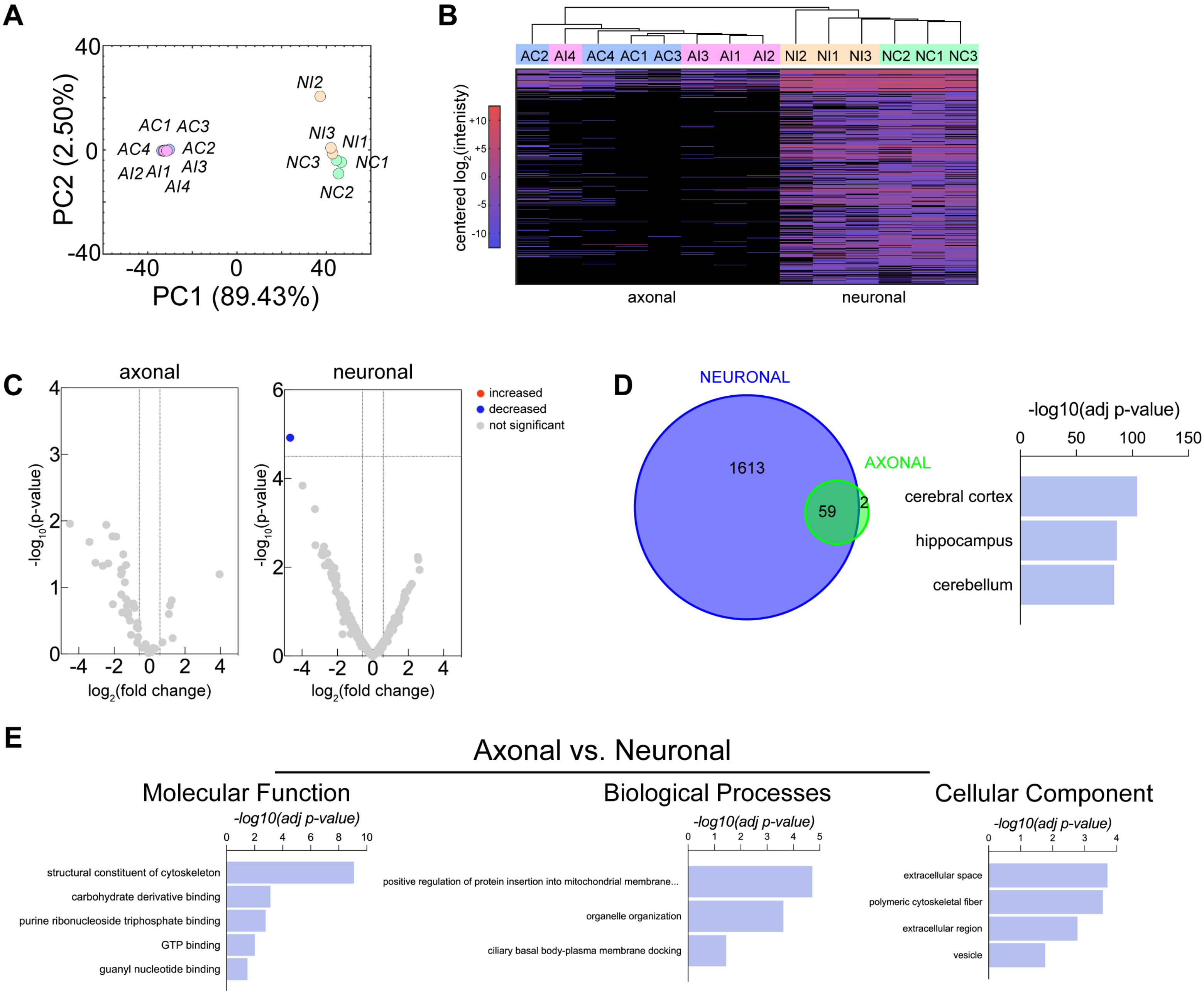

Figure S3. Axonal proteomic profiling before and immediately after injury, Related to Figure 4.
(A) PCA of axonal and neuronal fractions (A/N) in both control and injury treatments (C/I). n=4 axonal fractions, n=3 neuronal fractions.
(B) Heatmap of differentially expressed proteins.
(C) Volcano plots depicting the quantitative changes of injury compared with control treatments for the axonal and the neuronal fractions.
(D) Total number of proteins present in the neuronal and axonal fractions (left panel). Human Protein Atlas top terms of the enrichment analysis for the neuronal fraction proteins (right panel).
(E) Enrichment analysis of the axonal fraction proteins using as background the neuronal fraction proteins. Significant terms for each GO category.
