# Supplementary material for: Unraveling axonal mechanisms of traumatic brain injury
Source: Acta Neuropathol Commun. 2022 Sep 21;10:140. doi: 10.1186/s40478-022-01414-8 (PMC9494812; doi:10.1186/s40478-022-01414-8)
Supplement: Supplementary file 13 — Additional file 13: Fig. S4. Structural reorganization of the axonal cytoskeleton following injury, Related to Fig. 6. [file 40478_2022_1414_MOESM13_ESM.pdf]

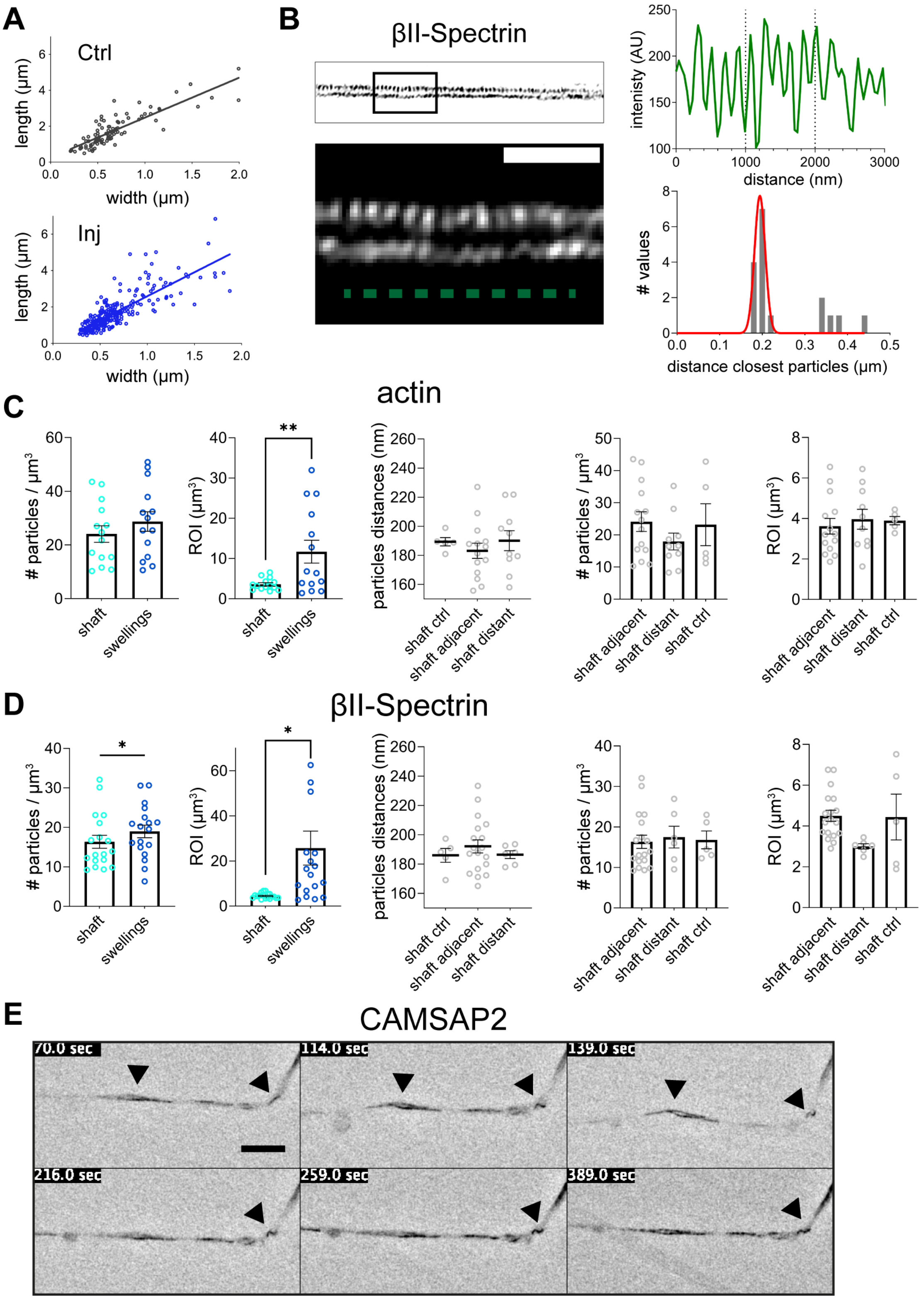

Figure S4. Structural reorganization of the axonal cytoskeleton following injury, Related to Figure 6.

(A) Correlation between the width and the length of the AS present in control and injured conditions in scanning microscopy images (control n=96, injury n=260). Linear regression for control and injured, $y=2.23x+0.2574$, $R2=0.73$ and $y=2.63x+0.04$, $R2=0.65$.

(B) Validation of the closest neighbour distance model to analyze distance between particles. Upper plot shows the profile of intensity through the axon long axis, showing inter-peaks distances of approximately 200 nm. Lower plot shows the closest neighbour distance analysis of the same axon, with a median of 200 nm. Scale Bar: 1 µm.

(C and D) Actin (C) and bII-Spectrin (D) particles densities and volumes for the different ROIs analyzed in Fig. 6E. Comparison of particles distances in shafts of axons that were not subjected to injury versus region of the shaft distant from the swelling versus region of the shaft adjacent to the swelling.

(E) Movie frames of CAMSAP2 transfected neurons showing the bending (arrowheads) of the CAMSAP2 filaments during and after injury (scale bar: 10 µm).

Data are mean±SEM (*$p<0.05$, **$p<0.01$). Statistical comparisons were performed using the student t-test and one way ANOVA.
