# Supplementary material for: Unraveling axonal mechanisms of traumatic brain injury
Source: Acta Neuropathol Commun. 2022 Sep 21;10:140. doi: 10.1186/s40478-022-01414-8 (PMC9494812; doi:10.1186/s40478-022-01414-8)
Supplement: Supplementary file 20 — Additional file 20. Key Resources Table: Table including antibodies, resources, kits, cells, and software with catalog numbers. [file 40478_2022_1414_MOESM20_ESM.docx]

KEY RESOURCES TABLE

| REAGENT or RESOURCE | SOURCE | IDENTIFIER |
| --- | --- | --- |
| Antibodies | | |
| eFluor450 mouse anti-CD15 | eBioscience | Cat#48-0159-42; RRID: AB_2016661 |
| APC mouse anti-CD24 | BioLegend | Cat#311132; RRID: AB_2566347 |
| PE mouse anti-CD44 | BioLegend | Cat#338808; RRID: AB_2076578 |
| PE mouse anti-CD184 | eBioscience | Cat#25-9999-42; RRID: AB_1659706 |
| eFluor450 mouse anti-CD15 | eBioscience | Cat#48-0566-42; RRID: AB_2574021 |
| PE mouse anti-CD140 | BioLegend | Cat#323508; RRID: AB_2565597 |
| AF488 mouse anti-O4 | R&D systems | Cat#FAB1326G |
| PE mouse anti-Nestin | BioLegend | Cat#656806; RRID: AB_2566382 |
| AF488 mouse anti-GFAP | eBioscience | Cat#53-9892-82; RRID: AB_10598515 |
| AF488 mouse anti-Sox2 | BioLegend | Cat#656110; RRID: AB_2563957 |
| PE rat anti-Ki67 | BioLegend | Cat#652404; RRID: AB_2561525 |
| eFluor450 mouse anti-CD15 | eBioscience | Cat#48-0159-42; RRID: AB_2016661 |
| APC mouse anti-CD24 | BioLegend | Cat#311132; RRID: AB_2566347 |
| PE mouse anti-CD44 | BioLegend | Cat#338808; RRID: AB_2076578 |
| PE mouse anti-CD184 | eBioscience | Cat#25-9999-42; RRID: AB_1659706 |
| eFluor450 mouse anti-CD56 | eBioscience | Cat#48-0566-42; RRID: AB_2574021 |
| PE mouse anti-CD140 | BioLegend | Cat#323508; RRID: AB_2565597 |
| AF488 mouse anti-O4 | R&D systems | Cat#FAB1326G |
| PE mouse anti-Nestin | BioLegend | Cat#656806; RRID: AB_2566382 |
| AF488 mouse anti-GFAP | eBioscience | Cat#53-9892-82; RRID: AB_10598515 |
| AF488 mouse anti-Sox2 | BioLegend | Cat#656110; RRID: AB_2563957 |
| PE rat anti-Ki67 | BioLegend | Cat#652404; RRID: AB_2561525 |
| mouse anti-Tuj1 | BioLegend | Cat#MMS-435P; RRID: AB_2313773 |
| mouse anti-NeuN | Millipore | Cat#MAB377; RRID: AB_2298772 |
| rabbit anti-Sox2 | CellSignaling | Cat#3579; RRID: AB_2195767 |
| goat anti-GFAP | Abcam | Cat#53554; RRID: AB_880202 |
| rabbit anti-Vimentin | Synaptic System | Cat#172002; RRID: AB_887887 |
| mouse anti-Map2 | Millipore | Cat#MAB3418; RRID: AB_11212326 |
| rabbit anti-Synapsin | Synaptic System | Cat#106002; RRID: AB_887804 |
| mouse anti-Nestin | Abcam | Cat#18102; RRID: AB_444246 |
| rabbit anti-CAMSAP2 | Atlas antibodies | Cat#HPA026511; RRID:AB_1852378 |
| mouse anti-BIISpectrin | BD | Cat#612563; RRID:AB_399854 |
| mouse anti-AcetylTUB | Sigma | Cat#T6793; RRID:AB_477585 |
| mouse anti-pNF-H (SMI31) | BioLegend | Cat#801601; RRID:AB_2564641 |
| AF488 goat anti-mouse | Life Technologies | Cat#A-11029; RRID: AB_138404 |
| AF488 goat anti-rabbit | Life Technologies | Cat#A-11034; RRID: AB_2576217 |
| AF555 donkey anti-mouse | Life Technologies | Cat#A-31570; RRID: AB_2536180 |
| AF555 donkey anti-rabbit | Life Technologies | Cat#A-31572; RRID: AB_162543 |
|  |  |  |
| Bacterial and virus strains | | |
| RLV-EF1a-mCherry-Mem | Vectalys | Cat#0026VCT |
| rLV-hSyn-GFP | Vectalys | N/A |
| rLV-hSyn-hAPP-GFP | Vectalys | N/A |
| rLV-hSyn-SYP-GFP | Vectalys | N/A |
| rLV-EF1a-AcGFP1-Mito | Vectalys | Cat#0017VCT |
|  |  |  |
| Biological samples |  |  |
|  |  |  |
| Chemicals, peptides, and recombinant proteins | | |
| KO DMEM/F12 | Gibco | Cat#12660 |
| StemPro Neural Supplement | Gibco | Cat#A10508 |
| Glutamax 100x | Gibco | Cat#35050 |
| bFGF | Gibco | Cat#PHG0021 |
| EGF | Gibco | Cat#PHG0314 |
| Matrigel | Corning | Cat#356231 |
| DMEM/F12 | Gibco | Cat#11330 |
| B27 50X | Gibco | Cat#17504 |
| N2 | Gibco | Cat#17502 |
| Laminin | Gibco | Cat#23017 |
| cAMP | Sigma | Cat#D0627 |
| Ascorbic acid | Sigma | Cat#A4403 |
| BDNF | Gibco | Cat#PHC7074 |
| GDNF | Gibco | Cat#PHC7041 |
| IGF | Peprotech | Cat#100-11 |
| Poly-ornithine | Sigma | Cat#P3655 |
| Accutase | Gibco | Cat#A11105 |
| Goat Serum | Sigma | Cat#G6767 |
| IC Fixation Buffer | eBioscience | Cat#00-8222-49 |
| Permeabilization Buffer | eBioscience | Cat#00-8333-56 |
| PFA | Chemcruz | Cat#sc-281692 |
| RIPA | Merck | Cat#20-188 |
| Phosphatase inhibitor | Sigma | Cat#P5726 |
| Proteinase inhibitor | Sigma | Cat#P8340 |
| Pluronic F-127 | Biotium | Cat#59005 |
| Triton X-100 | Sigma | Cat#T8787 |
| Ryanodine | Tocris | Cat#1329 |
| Nifedipine | Cayman | Cat#11106 |
| Gadolinium | Sigma | Cat#G7532 |
| CGP37157 | Tocris | Cat#1114 |
| Xestospongin C | Cayman | Cat#64950 |
| FBS | Sigma | Cat#9665 |
| EDTA | Invitrogen | Cat#AM9260G |
| Fluo-4, AM | Invitrogen | Cat#F14201 |
| WGA-633 | Invitrogen | Cat#W21404 |
| Dextran Cascade Blue (3KD) | Invitrogen | Cat#D7132 |
| Dextran Alexa Fluor 488 (10KD) | Invitrogen | Cat#D22910 |
| Dextran Tetramethylrodhamine (40 KD) | Invitrogen | Cat#D1842 |
| SBFI | Invitrogen | Cat#S1264 |
| PBFI | Invitrogen | Cat#P1267MP |
| FURA-2AM | Invitrogen | Cat#M1292 |
| DAPI | Roche | Cat#10236276001 |
| AlexaFluor 488 Phalloidin | Invitrogen | Cat#A12379 |
| TMRE | Invitrogen | Cat#T669 |
|  |  |  |
| Critical commercial assays | | |
| RNeasy Plus Micro kit | Qiagen | Cat#74034 |
| Transcriptor First Strand cDNA Synthesis Kit | Roche | Cat#04379012001 |
| Power SYBR® Green PCR Master Mix | Applied Biosystem | Cat#4368577 |
| ELISA kit Human Aβ1-40 | Novus Biologicals | Cat#NBP2-69909 |
| ELISA kit Human Aβ1-42 | Novus Biologicals | Cat#NBP2-69913 |
| BCA protein assay kit | Thermo | Cat#23227 |
|  |  |  |
| Deposited data | | |
|  |  |  |
| Experimental models: Cell lines | | |
| Human NSC | Gibco | Cat#51088 |
| HEK-293T | ATCC | Cat#CRL-3216 |
|  |  |  |
| Experimental models: Organisms/strains | | |
|  |  |  |
| Oligonucleotides | | |
|  |  |  |
| Recombinant DNA | | |
|  |  |  |
| Software and algorithms | | |
| FlowJo | BD | https://www.flowjo.com |
| AxIS v2.4 | Axion Biosystems | https://www.axionbiosystems.com |
| Zen (Blue Edition) | Carl Zeiss Microscopy | https://www.zeiss.com/microscopy/int/products/microscope-software/zen.html |
| IMARIS 64x v9 | Oxford Instruments | https://imaris.oxinst.com |
| ImageJ v1.53k | NIH | https://imagej.nih.gov/ij/ |
| Prism 9.3.0 | GraphPad Software | https://www.graphpad.com |
| Zeiss Zen 11 | Carl Zeiss Microscopy | https://www.zeiss.com/microscopy/int/products/microscope-software/zen.html |
|  |  |  |
| Other | | |
| Microfluidic Chambers uLP | Xona Microfluidics | Cat#uLP |
| Coverslips | Marienfeld | Cat#0107242 |
| Fluorescent Beads | BioLegend | Cat#422907 |
| MEA plate 48 MW | Axion | Cat#M768-tMEA-48MW |
| Mowiol | Sigma | Cat#81381 |
| Pillars (5 x 12 x 10) | RMS | N/A |
| u-Slide VI 0.4 | IBIDI | Cat#80606 |
| HSW NormJet 10 ml | VWR | Cat#53548-006 |
|  |  |  |
|  |  |  |
|  |  |  |
